# Combined Anti-Browning Mechanism of Pre-Cutting High Oxygen Treatment and Post-Cutting *Withania somnifera* Extract Treatment on Fresh-Cut Apples

**DOI:** 10.3390/foods15152673

**Published:** 2026-07-29

**Authors:** Xiangzheng Yang, Huan Lian, Shunxin Qin, Di Wu

**Affiliations:** 1 College of Agriculture & Biotechnology, Zhejiang University, Hangzhou 310058, China; yangxiangzheng318@163.com; 2 Jinan Fruit Research Institute, All China Federation of Supply and Marketing Cooperatives, Jinan 250014, China; lianhuan0326@163.com (H.L.); qsx0001999@163.com (S.Q.)

**Keywords:** anti-browning mechanism, ashwagandha extract, browning inhibition, fresh-cut apple, high oxygen pre-treatment, proteomics

## Abstract

Fresh-cut apples are highly prone to browning during processing and storage. This long-standing problem will cause huge economic losses and restrict the commercial development potential of the fresh-cut fruit industry. In this study, Fuji apples were used as test materials to explore the inhibitory effect and anti-oxidant mechanism of pre-cutting high oxygen treatment (90% O_2_, 3 h) combined with post-cutting *Withania somnifera* extract immersion on the browning of fresh-cut apples. Through the determination of physiological and biochemical indexes and proteomics analysis, the effects of different treatments on the nutritional quality, reactive oxygen species metabolism, membrane lipid peroxidation, anti-oxidant enzyme activity and related protein expression of fresh-cut apples were systematically evaluated. The results showed that after 14 days of storage, the combined treatment group had the lowest browning index of 41.29% and the highest whiteness index of 60.35%, and its color protection effect was significantly better than that of single high oxygen treatment or single extract treatment. The combined treatment could increase the total phenol content to 6.45 mg/g after 7 days of storage, and increase the DPPH free radical scavenging rate to 0.60% after 14 days of storage, significantly enhancing the anti-oxidant capacity. Meanwhile, the combined treatment could maintain higher activities of superoxide dismutase (SOD), catalase (CAT) and peroxidase (POD), and maintain the malondialdehyde content at a low level of 0.06 nmol/g after 14 days of storage, effectively delaying the process of membrane lipid peroxidation. However, adverse results were also observed in the study, including the rebound of superoxide anion level (28.16 nmol/g) after 14 days of storage, the highest accumulation of H_2_O_2_ during the whole storage period, and the significant increase in polyphenol oxidase (PPO) activity (151.14 IU/L) after 14 days of storage, indicating an elevated browning risk under prolonged storage. Proteomics analysis revealed that the combined treatment regulated multiple metabolic pathways, including the up-regulating of glutathione metabolism and pentose phosphate pathway to enhance reactive oxygen species scavenging capacity, as well as the modulation of fatty acid metabolism to preserve cell membrane integrity. Despite the above limitations, the overall browning inhibitory effect of the combined treatment is still better than that of a single treatment, but its pro-oxidant effect and long-term reactive oxygen species regulation effect still need to be further optimized. In summary, pre-cutting high oxygen treatment combined with post-cutting *Withania somnifera* extract treatment is an effective anti-browning strategy for fresh-cut apples, which can provide a theoretical basis for the development of natural and efficient fresh-cut fruit and vegetable preservation technology.

## 1. Introduction

Apples are rich in vitamins, dietary fiber and essential minerals, and have preventive effects on chronic diseases, so they are deeply loved by consumers. However, enzymatic browning remains a critical challenge limiting the shelf life and commercial value of fresh-cut apples [1,2]. Mechanical damage will destroy the cell compartmentalization structure, induce enzymatic oxidation of phenolic substances and reactive oxygen species burst, and then accelerate membrane lipid peroxidation and protein degradation, eventually leading to the deterioration of appearance and texture quality of fresh-cut products [3,4,5]. At present, the academic community has developed a variety of preservation strategies to alleviate the above problems, which can be roughly divided into three categories: physical methods (such as modified atmosphere packaging and ultra-high pressure treatment) [6,7], chemical methods (anti-oxidant dips) [8,9,10] and biological methods (natural extracts) [11,12,13,14]. Importantly, single-technology approaches struggle to simultaneously achieve efficient anti-browning, nutritional quality maintenance, and food safety assurance.

High oxygen treatment has been applied to a variety of fruits and vegetables such as apples [15], strawberries [16], carrots [17], etc., with wide applicability, and its effects of inhibiting browning and delaying yellowing are particularly prominent. For example, short-term high oxygen pre-stimulation (75~95% O_2_) can effectively delay the browning of fresh-cut apples and maintain their bright surface color [15]. Meanwhile, pre-treatment with 50% O_2_ + 50% CO_2_ can significantly delay the yellowing process of fresh-cut broccoli and extend its shelf life [18]. The underlying mechanism is two-fold: moderate HO acts as mild oxidative stress that directly inhibits PPO and POD activities via feedback inhibition or substrate competition [15]; simultaneously, it regulates energy metabolism, ROS balance, membrane lipid metabolism, and phenolic synthesis at the systemic level to enhance tissue tolerance to browning [15,18].

Among the potential alternative natural sources being screened, ashwagandha (*Withania somnifera*) has become a promising candidate material due to its excellent anti-oxidant capacity. Its active components include withanolides, alkaloids, saponins, phenolic acids and flavonoids [19], among which withaferin A is the most widely studied and most active compound, and the unsaturated ketone and lactone ring in its molecular structure are the key functional groups for biological activity [20]. The anti-oxidant mechanisms of ashwagandha operate via two pathways: first, it can directly scavenge free radicals: the unsaturated bonds and hydroxyl groups in the withanolide structure have strong free radical scavenging ability, which can effectively quench a variety of reactive oxygen species, including 1,1-diphenyl-2-picrylhydrazyl (DPPH), superoxide anion (O_2_^−^·) and hydrogen peroxide (H_2_O_2_) [21]; second, it can activate the endogenous anti-oxidant system: existing studies have shown that ashwagandha extract can up-regulate the expression and activity of anti-oxidant enzymes such as SOD, CAT, glutathione peroxidase (GPx), so as to improve the intrinsic anti-oxidant defense capacity of cells [22]. 

The combination of high oxygen treatment and ashwagandha extract treatment can realize the combined protection mode of “stress-induced activation + exogenous supplementation”, exert a more lasting and comprehensive anti-browning effect, reduce the required intensity of a single treatment, and make up for the defect that the effect of single high oxygen treatment decays with time. At present, few studies have integrated pre-cutting induced resistance with post-cutting immediate exogenous defense, and the interplay between these two strategies remains largely unexplored. Therefore, filling this research gap is of great significance for the development of the next generation of efficient, sustainable and consumer-popular fresh-cut fruit and vegetable preservation technology.

This study aims to systematically explore the anti-browning effect and mechanism of pre-cutting high oxygen treatment combined with post-cutting ashwagandha extract treatment. The specific research objectives are: to clarify the inhibitory effect of combined treatment on the browning of fresh-cut apples; to elucidate the physiological and biochemical mechanism of the combined effect from the perspectives of enzyme activity, phenolic metabolism, reactive oxygen species metabolism and membrane lipid peroxidation; and to reveal the molecular network of joint regulation through proteomics analysis. This study is expected to provide theoretical basis and technical support for the development of new, green and efficient fresh-cut apple preservation technology.

## 2. Materials and Methods

### 2.1. Test Materials

Fuji apples (Malus domestica Borkh. cv. Fuji) of uniform size (200~250 g), commercial maturity, and free from defects were harvested from a commercial orchard in Yantai, China. Fruits were immediately stored at 4 °C ± 0.5 °C with relative humidity of 90~95% after screening, and used within 3 days of harvest.

Ashwagandha plant powder (using dried roots as raw material, and ethanol as the extraction solvent for crude extraction, the obtained extract is concentrated, dried and sieved through a 100-mesh sieve to obtain brown powder) was purchased from Xi’an Ruihe Biological Engineering Technology Co., Ltd. (Xi’an, China). Methanol, ethanol, Folin–Ciocalteu reagent, sodium carbonate, gallic acid, thiobarbituric acid, trichloroacetic acid, 1,1-diphenyl-2-picrylhydrazyl (DPPH), polyvinylpyrrolidone, catechol, guaiacol, hydrogen peroxide were all purchased from Sinopharm Chemical Reagent Co., Ltd. (Shanghai, China), and all chemical reagents used were of analytical grade.

### 2.2. Preparation of Plant Extracts

Mix ashwagandha (*Withania somnifera*) plant powder with distilled water at a ratio of 1:20 (g/mL), and extract for 2.5 h. Then use ST 8R high-speed centrifuge (Thermo Fisher Scientific, Waltham, MA, USA) to centrifuge at 4 °C and 8000 r/min for 20 min, collect the supernatant to obtain the plant extract, and store it at 4 °C for later use.

### 2.3. Fresh-Keeping Treatment of Fresh-Cut Apples

Soak apples in 3% (mass concentration) hydrogen peroxide solution for 3 min, then air dry naturally. Oxygen treatment step: Place 20 apple samples in a closed glass chamber (volume 20 L, wall thickness 5 mm); first purge with high-purity nitrogen (N_2_ ≥ 99.999%) to create an anaerobic environment, then introduce medical-grade oxygen (O_2_ ≥ 99.95%) until the oxygen concentration reaches 90% (the rest is balanced by nitrogen). Place the samples in this high oxygen environment and treat them at 25 ± 1 °C for 3 h, while the control group (CK) samples are treated simultaneously in a conventional environment (21% O_2_). Use the calibrated YGA2200 O_2_/CO_2_ analyzer (Beijing Yangguang Yishida Technology Co., Ltd., Beijing, China) to monitor the indoor gas composition in real time. After the high oxygen treatment, cut the treated apples into 8 equal pieces, remove the core, then immerse them in 20-fold diluted ashwagandha aqueous extract (prepared with distilled water) at 4 °C for 3 min. After the immersion treatment, wipe off the surface moisture, use an automatic vacuum packaging machine (Jining Hongsheng’an Machinery Co., Ltd., Jining, China) to pack the samples into polyethylene bags (thickness 0.7 mm, specification 150 mm × 100 mm) for vacuum packaging, store them at 4 °C with relative humidity of 90~95%, and sample them on the 0th, 7th and 14th day of storage respectively to determine relevant indexes. This study sets different treatment groups: a control group, a single high oxygen treatment group, a single ashwagandha extract immersion treatment group, and a high oxygen + extract immersion combined treatment group, named CK, HO, WS, and CT respectively.

### 2.4. Determination of Physiological Indexes

#### 2.4.1. Appearance Phenotype

Take photos to record the changes of appearance phenotype of the fresh-cut apples.

#### 2.4.2. Browning Index (BI) and Whiteness Index (WI)

Use a CR-400 precision color difference meter (Konica Minolta, Inc., Tokyo, Japan) to determine the surface color of the apple slices to obtain L*, a*, b* values, then calculate the browning index (BI) and whiteness index (WI) with the following formulas:

(1) $$BI=\frac{100\times \left(\frac{a^{*}\;+\;1.75L^{*}}{5.645L^{*}\;+\;a^{*}-3.012b^{*}}-0.31\right)}{0.172}$$

(2) $$WI=100-\sqrt{(100-L^{*}{)}^{2}+a^{*2}+b^{*2}}$$

#### 2.4.3. Soluble Solids Content and Titratable Acidity

Use PAL-1 digital refractometer (ATAGO CO., LTD., Tokyo, Japan) to determine the soluble solids content, and the titratable acidity is determined by the acid–base titration method.

### 2.5. Determination of Anti-Oxidant Indexes

#### 2.5.1. Total Phenol Content

The total phenol content is determined according to the method reported by Hu et al. [23], and expressed as gallic acid equivalents (mg g^−1^). 

#### 2.5.2. Determination of Malondialdehyde Content

Malondialdehyde content is determined with reference to the method reported by Feng et al. [24].

#### 2.5.3. DPPH Free Radical Scavenging Rate

The DPPH free radical scavenging rate is determined with reference to the method reported by Shi et al. [25].

#### 2.5.4. Contents of Superoxide Anion (O_2_^−^·) and Hydrogen Peroxide (H_2_O_2_)

The contents of O_2_^−^· and H_2_O_2_ are determined with an O_2_^−^· detection kit and H_2_O_2_ detection kit (Jiangsu Aidisheng Biotechnology Co., Ltd., Yancheng, China) respectively. The O_2_^−^· content is expressed by the amount of O_2_^−^· produced per gram of apple tissue, with the unit of nmol/g. Similarly, the H_2_O_2_ content is expressed by the amount of H_2_O_2_ produced per gram of apple tissue, with the unit of μmol/g.

#### 2.5.5. Enzyme Activity Assays

PPO (polyphenol oxidase) activity, POD (peroxidase) activity, CAT (catalase) activity, SOD (superoxide dismutase) activity and PAL (phenylalanine ammonia-lyase) activity are determined with a plant ELISA kit (Jiangsu Jingmei Biotechnology Co., Ltd., Yancheng, China) following the manufacturer’s instructions.

### 2.6. High-Throughput Proteomics

To further clarify the regulatory mechanism of different treatments on the browning of fresh-cut apples, the control group (CK), pre-cutting high oxygen treatment group (HO), post-cutting *Withania somnifera* extract soaking treatment group (WS) and combined treatment group (pre-cutting high oxygen + post-cutting extract soaking, CT) on the 7th day of storage are selected for DIA proteomic analysis, with 3 biological replicates set for each group. Based on the change trends of browning index and anti-oxidant enzyme activity from the preceding experimental data, the differences among treatment groups on the 7th day of storage are the most significant, which is the key time point for analyzing the protein regulatory network.

Protein extraction, tryptic digestion, peptide desalting, and quantification are performed following standard procedures (detailed in Supplementary Materials). DIA mass spectrometry is performed on an Astral mass spectrometer (Thermo Scientific) coupled with a Vanquish Neo nano-LC system (LC gradient and MS parameters provided in Supplementary Table S1).

### 2.7. Data Processing and Analysis

All data are obtained from 3 replicates, and the final results are expressed as mean ± standard deviation (Mean ± SD). Origin 8.0 software is used to draw charts, and SPSS Statistics 24 software is used for statistical analysis of data. Duncan’s multiple range test is used to analyze the significance of differences among multiple groups of samples, and *p* < 0.05 indicates that the difference is statistically significant.

## 3. Results and Analysis

### 3.1. Changes in Appearance Phenotype of Fresh-Cut Apples

**Figure 1:**
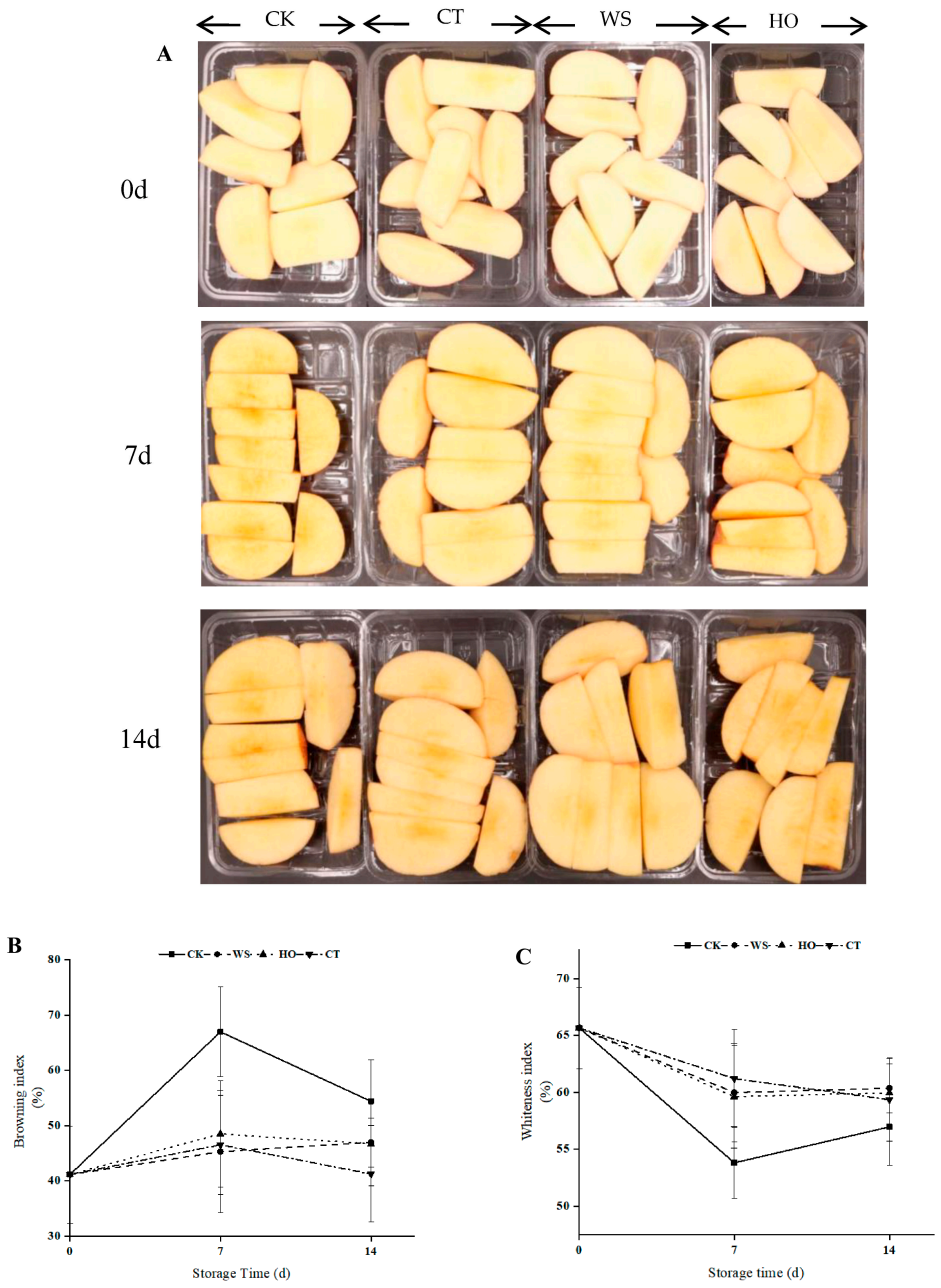
The effect of high oxygen combined with *Withania somnifera* extract treatment on the appearance phenotype (**A**), browning index (BI) (**B**) and whiteness index (WI) (**C**) of fresh-cut apples. Values are expressed as mean ± SD (n = 6). Vertical bars represent standard deviation (**B**,**C**).

The anti-browning effects of different treatments on fresh-cut apples are shown in Figure 1. On day 0 of storage, the cut surfaces of all treatment groups are fresh milky white or light yellow, with no visible browning. After 7 days of storage, the cut surface of the control group (CK) has obvious light brown patches, dark edges, and the browning area accounts for about 30~40%, showing typical enzymatic browning characteristics. In contrast, the CT and WS groups have lower browning degrees, most of the cut surfaces still remain light color, and only the edges show light brown. Among them, the CT group has the best performance, maintaining surface color nearly unchanged from day 0, with high brightness and uniformity. After 14 days of storage, the browning of the CK group is further intensified; the cut surface turns dark brown, accompanied by water loss and shrinkage, and local areas have a wet and sticky feeling, suggesting that cell structure damage and microbial activity may have aggravated quality deterioration. The browning area of the HO and WS groups expands to 50~60%, and the appearance color is darker. However, the CT group still only shows mild to moderate browning, showing a continuous and stable color protection effect. The above results show that the effect of CT treatment is the most stable, which may delay browning by effectively inhibiting polyphenol oxidase activity, maintaining cell membrane integrity, and reducing the contact between phenolic substances and enzymes [7]. The sticky feeling in the control group in the later stage indicates that microbial growth has become a secondary factor of quality deterioration, indicating that long-term storage needs to be combined with antibacterial measures.

Browning index (BI) and whiteness index (WI) were used to quantitatively evaluate surface discoloration [15]. From the perspective of browning index (BI) (Figure 1B), the BI values of all treatment groups gradually increase with the extension of storage time. On day 0, the BI value of all groups is 41.19%. On day 7, the BI of the CK group rises sharply to 66.98%, while the BI values of the CT group (46.57%), WS group (45.32%) and HO group (48.54%) are still significantly lower, which are 20.41, 21.66 and 18.44 percentage points lower than CK respectively, indicating that these three treatments can effectively inhibit browning. On day 14, the BI of the CK group rises to 54.41%, the WS group and HO group both reach 47.0%, while the CT group has the lowest BI of 41.29%, which is closest to the initial level, indicating that the CT treatment can maintain a stable browning inhibitory effect during the entire 14-day storage period. From the perspective of whiteness index (WI) (Figure 1C), the WI values of all treatment groups continue to decrease with the extension of storage time. On day 0, the WI value of all groups is 65.65%. On day 7, the WI of the CK group drops to 53.81% (a decrease of 11.84 percentage points), while the WI of the CT group (61.20%), WS group (59.97%) and HO group (59.62%) remains at a high level, among which the CT group has the highest WI. On day 14, the WI of the CK group further drops to 56.5%, the WS group and HO group are both 59.0%, while the CT group still maintains the highest WI value (60.35%), with the smallest deviation from the initial level, indicating that its effect in maintaining the whiteness of the cut surface is better. The above results show that the CT treatment is the treatment with the best effect in inhibiting browning and maintaining surface whiteness among all test groups. This excellent performance may be because it can more effectively inhibit polyphenol oxidase activity and maintain the integrity of cell membrane structure, thereby reducing the contact between phenolic substrates and enzymes and delaying the enzymatic browning process. In addition, CT treatment can still maintain a high whiteness index during long-term storage, indicating that it also has a certain inhibitory effect on non-enzymatic browning.

### 3.2. Changes in Soluble Solids and Titratable Acid Contents of Fresh-Cut Apples

**Figure 2:**
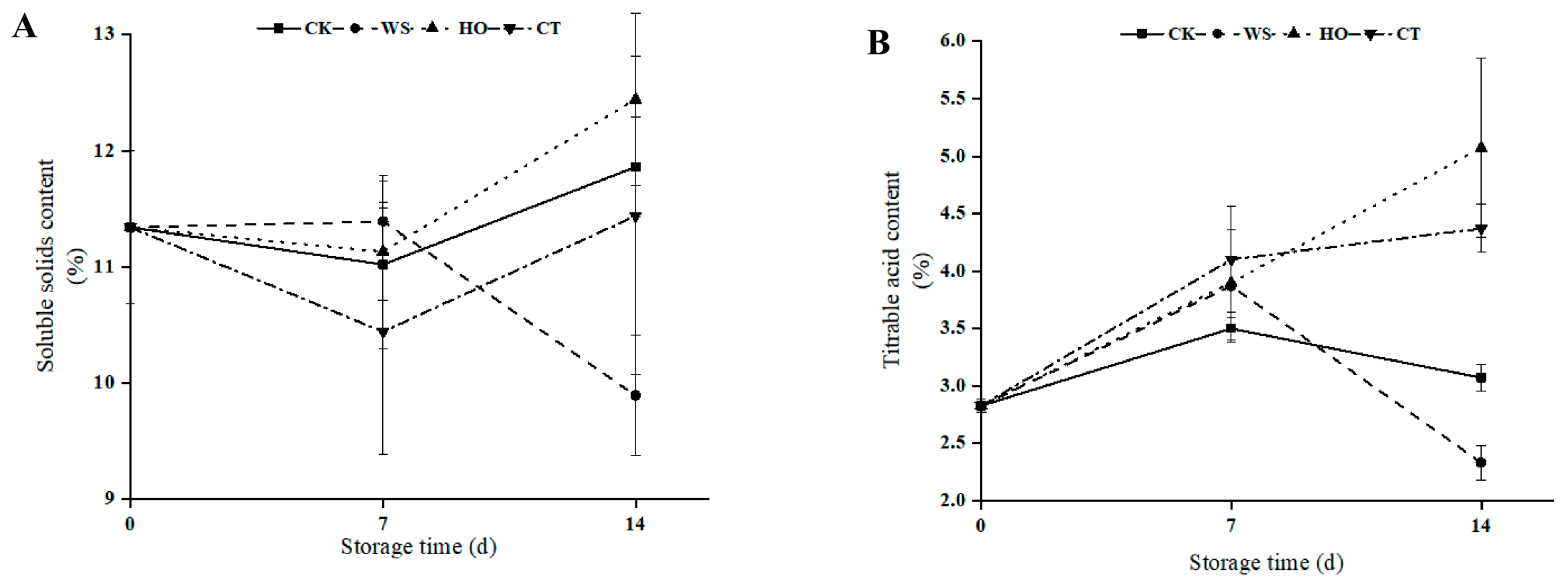
Effect of high oxygen combined with *Withania somnifera* anti-oxidant treatment on soluble solids content (**A**) and titratable acid content (**B**) of fresh-cut apples. Values are expressed as mean ± SD (n = 6). Vertical bars represent standard deviation.

As shown in Figure 2, on day 0 of storage, the initial total soluble solids (TSS) and titratable acid (TA) contents of fresh-cut apples in all groups are about 11.34% and 2.90 (calculated as malic acid) respectively, with no significant difference between groups (*p* > 0.05). On day 7 of storage, the TSS of all groups decreases slightly compared with that on day 0, but the decrease range is small; at the same time, the TA content of all groups increases, among which the HO group has the highest value (4.05%). On day 14 of storage, the TSS of the HO group rises significantly to 12.44%, which is the highest value among all treatment groups (*p* > 0.05), which may be because high oxygen pre-treatment promotes the conversion of starch to soluble sugar, or the concentration effect caused by surface dehydration [26]; the TSS of the CT group has almost no change compared with that on day 0, showing the most stable performance. In terms of titratable acid, on day 14 of storage, the TA content of the CK group and WS group decreases compared with that on day 7, while the TA content of the HO group and CT group continues to rise, and is significantly higher than that of the CK group (*p* > 0.05), indicating that single high oxygen pre-treatment (HO) and high oxygen combined with ashwagandha extract treatment (CT) can effectively delay the degradation of titratable acid in fresh-cut apples in the late storage period [27]. In conclusion, the CT group has the best overall performance in maintaining TSS stability and delaying TA degradation, which proves that pre-cutting high oxygen treatment combined with post-cutting ashwagandha extract treatment has a beneficial effect on the nutritional quality preservation of fresh-cut apples.

### 3.3. Changes in Anti-Oxidant Properties and Reactive Oxygen Species Metabolism of Fresh-Cut Apples

**Figure 3:**
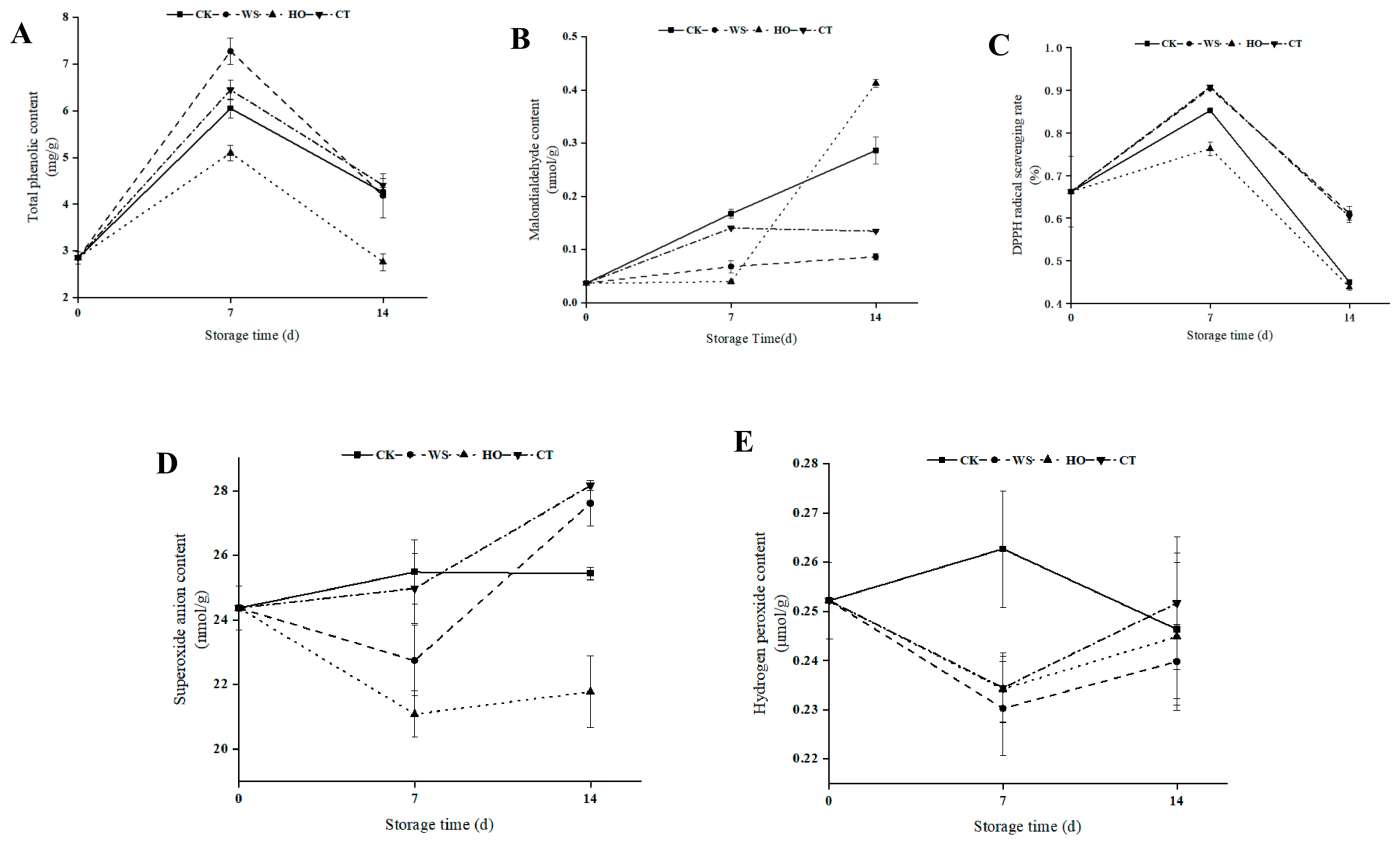
Effect of high oxygen combined with *Withania somnifera* anti-oxidant treatment on total phenol content (**A**), malondialdehyde content (**B**) and DPPH free radical scavenging rate (**C**), superoxide anion content (**D**) and hydrogen peroxide content (**E**) of fresh-cut apples. Values are expressed as mean ± SD (n = 3). Vertical bars represent standard deviation.

As shown in Figure 3A, with the extension of storage time, the total phenol content of fresh-cut apples generally shows a trend of first increasing and then decreasing. In the early stage of storage, the cutting operation destroys the compartmentalization structure of cells. On the one hand, it activates enzymes related to phenol synthesis; on the other hand, it induces tissue to produce stress response, so that the total phenol content accumulates rapidly and reaches the peak. In the late stage of storage, tissue aging intensifies, the phenol synthesis ability decreases, and the remaining phenolic substrates are continuously oxidized and consumed under the catalysis of polyphenol oxidase (PPO), leading to the gradual decrease in total phenol content [28,29]. On day 7 of storage, the total phenol content of the WS group and CT group is significantly higher than that of the control group (*p* < 0.05), reaching 7.28 mg/g and 6.45 mg/g respectively, indicating that these two treatments can more effectively delay the degradation of phenols or promote the retention of phenols, so as to maintain higher anti-oxidant capacity of the tissue. On day 14 of storage, the total phenol content of the HO group drops to the lowest value of 2.76 mg/g, indicating that this treatment may accelerate the oxidative consumption of phenolic compounds or inhibit their synthesis, which is not conducive to the maintenance of the storage quality of fresh-cut apples. The above results show that the WS and CT treatment groups can delay the oxidative browning and degradation of phenolic compounds by reducing polyphenol oxidase (PPO) activity, regulating cell compartmentalization structure or improving anti-oxidant enzyme activity [30].

Malondialdehyde (MDA) is an important product of membrane lipid peroxidation, and its content can reflect the damage degree of the cell membrane and the aging process of fresh-cut apples during storage [31]. As shown in Figure 3B, with the extension of storage time, the MDA content of all treatment groups shows an increasing trend, indicating that the degree of membrane lipid peroxidation of fresh-cut apples continues to intensify during storage, and the cell membrane damage gradually worsens. On day 7 of storage, the increase range of the control group (CK) is the most significant, and the MDA value reaches 0.17 nmol/g, while the HO group has the smallest increase range, with an MDA value of only 0.04 nmol/g. On day 14 of storage, the MDA content of the WS group slowly rises to 0.09 nmol/g, maintaining a relatively low level overall. The MDA content of the CT group decreases slightly between day 7 and day 14; notably, the MDA content of the HO group surged sharply to 0.41 nmol/g on day 14, far exceeding that of the other treatment groups. The above differences are mainly attributed to the varying capabilities of each treatment in regulating membrane lipid peroxidation. In the early storage period, the CK group exhibited the largest increase in MDA, indicating that the CK group is more sensitive to mechanical damage and oxidative stress under short-term storage. The HO group showed the smallest increase, indicating that high oxygen treatment can effectively delay MDA production in the early stage of storage, showing a certain oxidative tolerance. In the later storage period, the WS group showed a slow increase, while the CT group exhibited a slight decline, reflecting their superior sustained anti-oxidant capacity. However, the MDA content of the HO group increased sharply on day 14, which is much higher than that of other treatment groups, indicating that the membrane lipid peroxidation process of this treatment is out of control under long-term storage, the cell membrane structure is seriously damaged, and there is a lack of an effective anti-oxidant protection mechanism. The above results show that the WS and CT groups have the most stable performance and can effectively maintain the integrity of cell membrane, which is speculated to be related to their stronger reactive oxygen species scavenging ability or higher anti-oxidant enzyme activity [32]. Although the HO group performs well in short-term storage, its anti-oxidant protection mechanism is insufficient under long-term storage, which instead leads to the intensification of membrane lipid peroxidation [33].

DPPH free radical scavenging activity is an important indicator for evaluating anti-oxidant capacity. As shown in Figure 3C, the effects of different treatments on the DPPH free radical scavenging activity of fresh-cut apples generally show a trend of first increasing and then decreasing. In the early stage of storage, the DPPH scavenging activity of all treatment groups increases, among which the WS group and CT group have the largest increase, while the CK group and HO group have relatively lower increases. This initial increase can be attributed to the defense response induced by cutting injury and the activation of the anti-oxidant enzyme system, which jointly promote the improvement of tissue anti-oxidant capacity [32]. With the extension of storage time, by the 14th day, the DPPH scavenging activity of all treatment groups decreases significantly. Among them, the CK group and HO group have the largest decrease, and the CT group and WS group decrease to 0.60% and 0.61% respectively, both higher than the CK group. Although these values are lower than those in the early stage of storage, they are still higher than those of the CK group, indicating that their protective effect in the later stage is better. The above results show that there are significant differences in the effects of different treatments on maintaining anti-oxidant capacity in the late storage period. HO treatment cannot effectively maintain anti-oxidant capacity under long-term high oxygen environment, and long-term exposure to a high oxygen environment will lead to the accumulation of oxidative damage, accelerate the consumption of anti-oxidant substances or inhibit their synthesis, thus promoting the accelerated decline of anti-oxidant capacity [34]. In contrast, the WS and CT treatments activate and maintain the endogenous defense system through exogenous anti-oxidants, effectively delaying the decline of the anti-oxidant capacity of fresh-cut apples, and can play a stable protective role even under high oxygen conditions [35].

Reactive oxygen species (ROS) in plant cells are mainly produced by mitochondria, including superoxide anion free radicals and H_2_O_2_, and their generation and scavenging processes are in a dynamic balance [36,37]. As shown in Figure 3D, with the extension of storage time, the superoxide anion content of all treatments generally shows a trend of first decreasing and then increasing. On day 0 of storage, the initial content of all treatment groups is 24.36 nmol/g. On day 7 of storage, the superoxide anion contents of the WS group, HO group and CT group are all lower than that of the CK group, which are 22.74 nmol/g, 21.08 nmol/g and 24.97 nmol/g respectively. Among them, the HO group has the largest decrease range, indicating that moderate high oxygen treatment can effectively inhibit the generation of superoxide anions or promote their scavenging in a short term. On day 14 of storage, the HO treatment has the lowest superoxide anion content of 21.77 nmol/g, which is consistent with the trend of continuous relief of oxidative stress. However, the superoxide anion contents of the CT group (28.16 nmol/g) and WS group (27.61 nmol/g) are higher than that of the CK group, indicating that these two treatments will aggravate oxidative stress under long-term storage. The higher levels observed in the CT and WS treatments indicate that the anti-oxidant treatment and its combined effect with high oxygen under the experimental conditions will aggravate oxidative stress, which may be because exogenous anti-oxidants produce pro-oxidant effects at specific concentrations or under specific environmental conditions [38]; it may also be that excessive scavenging of reactive oxygen species (ROS) signals inhibits the up-regulation of the endogenous defense system, leading to impaired adaptive response when stress persists. In the CT treatment, high oxygen may accelerate the autoxidation of anti-oxidants, leading to worse effect [39]. However, it is worth noting that the HO treatment does not show this late increase phenomenon, indicating that the pro-oxidant transition is treatment-specific and not a universal result of anti-oxidant application. The above results show that not all anti-oxidant treatments can maintain beneficial effects in long-term storage, and the interaction between treatment methods and storage duration deserves further attention. Particularly importantly, the data show a temporal contradiction: the treatment that seems effective on day 7 produces negative effects on day 14 instead, which indicates that caution is needed when extrapolating short-term anti-oxidant effects to long-term storage scenarios.

Hydrogen peroxide (H_2_O_2_) is an important reactive oxygen species produced by plant tissues under stress conditions, and its accumulation level can directly reflect the oxidative stress level of fresh-cut apples as well as the regulatory capacity of the anti-oxidant system [40]. As shown in Figure 3E, with the extension of storage time, there are significant differences in the effects of different treatments on hydrogen peroxide content. The H_2_O_2_ content of the CK group shows a trend of first increasing and then decreasing, while the H_2_O_2_ content of other treatments shows a trend of first decreasing and then increasing, indicating that high oxygen combined with ashwagandha (*Withania somnifera*) extract treatment can effectively reduce H_2_O_2_ levels in the early stage of storage, while CK treatment shows the characteristic of delayed response. The hydrogen peroxide content of the WS treatment group is always the lowest during the whole storage period, indicating that its inhibitory effect on oxidative stress is the most stable among all treatments. In contrast, the CT treatment has the highest hydrogen peroxide content, even exceeding that of the control group, indicating that the combined treatment unexpectedly leads to an abnormal increase in hydrogen peroxide levels. This finding is of particular concern, as it runs counter to the anticipated complementary effects of high oxygen combined with exogenous anti-oxidants. This may be because the high oxygen environment accelerates the autoxidation of exogenous anti-oxidants, converting them into pro-oxidants, and at the same time inhibits the activity of hydrogen-peroxide-scavenging enzymes, leading to an antagonistic effect of the combined treatment [41]. However, there is a lack of direct evidence for pro-oxidant conversion or enzyme inhibition in this specific system, so this explanation remains speculative. In addition, there are differences in the responses of superoxide anion and hydrogen peroxide: the WS treatment shows poor performance in superoxide anion but the best performance in H_2_O_2_, which highlights the complexity of ROS dynamics, indicating that different treatments may disturb different branches of the ROS metabolic network. These inconsistencies should be recognized rather than ignored, as they indicate that the relationship between treatment, ROS and storage time is not linear and cannot be fully reflected by a single ROS marker.

### 3.4. Changes in Anti-Oxidant Enzyme Activity of Fresh-Cut Apples

**Figure 4:**
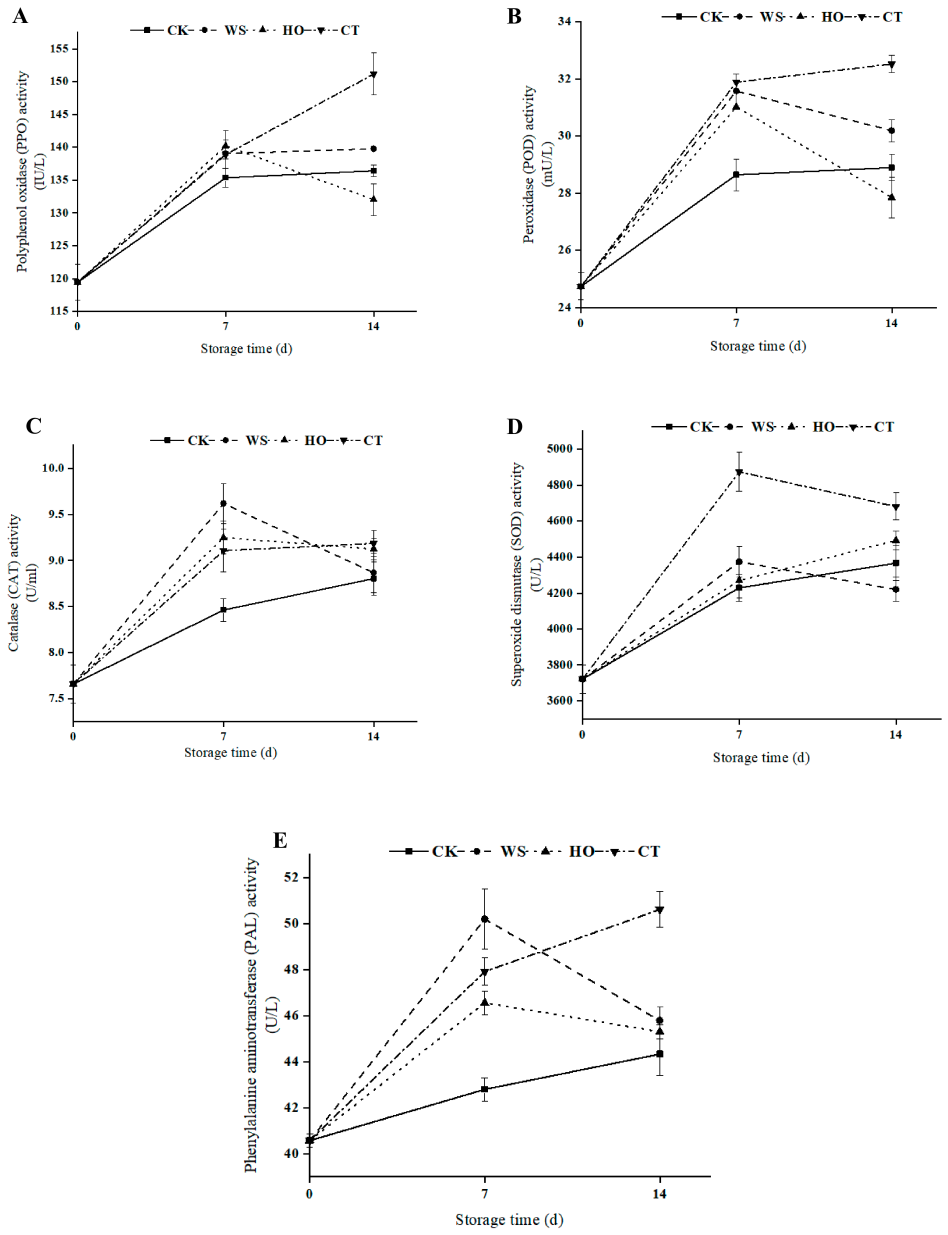
The effect of high oxygen combined with *Withania somnifera* anti-oxidant on the polyphenol oxidase (PPO) (**A**), peroxidase (POD) (**B**), catalase (CAT) (**C**), superoxide dismutase (SOD) (**D**) and phenylalanine ammonia-lyase (**E**) activities of fresh-cut apples. Note: Values are expressed as mean ± SD (n = 3). Vertical bars represent standard deviation.

PPO and POD are key enzymes in the enzymatic browning process of phenolic substances [42]. There are significant differences in the effects of different treatments on PPO and POD activities (Figure 4A,B). On day 0 of storage, the initial PPO activity of all groups is 119.4 IU/L, and the initial POD activity is 24.74 mL/L. With the extension of storage time, the PPO and POD activities of all treatment groups show an upward trend. In terms of PPO activity, on day 7 of storage, the PPO activity of the control group rises to 135.35 IU/L, while the PPO activities of the HO, WS and CT groups are all higher than that of the control group, indicating that all three treatments further induce or activate PPO to a certain extent. On day 14 of storage, the PPO activity of the control group is maintained at 136.42 IU/L, that of the WS group is 139.78 IU/L, that of the HO group drops to 132.03 IU/L, while that of the CT group further rises to 151.14 IU/L, which is significantly higher than that of the control group and other treatment groups (*p* < 0.05). The continuous increase in PPO activity in the CT group means higher browning risk, which is contrary to the expected anti-browning effect of this treatment. As for POD activity, on day 7 of storage, the POD activities of the HO, WS and CT groups are all higher than that of the control group, indicating that all three treatments activate POD to varying degrees in the early storage period, which is consistent with the induced anti-oxidant stress response. On day 14, the POD activities of the WS and CT groups are the highest, reaching 30.19 mL/L and 32.52 mL/L respectively, which are significantly higher than those of the control group (28.90 mL/L) and HO group (27.85 mL/L) (*p* < 0.05), indicating that these two treatments maintain higher POD activity in the late storage period, which is theoretically helpful to scavenge accumulated H_2_O_2_ and delay membrane lipid peroxidation [15]. In contrast, the POD activity of the control group changes slowly and lacks effective regulation, indicating that its reactive oxygen species (ROS) scavenging capacity is relatively weak [43]. The results show that WS treatment is the most conducive to balancing stress response and browning risk: it maintains relatively high POD activity to improve anti-oxidant capacity, while avoiding excessive increase in PPO, and has the best regulatory effect on enzyme activity. Although the CT treatment maintains high POD activity in the later stage, its PPO activity increases abnormally, leading to the highest browning risk. The PPO and POD activities of the HO treatment decrease significantly in the late storage period, reflecting metabolic decline and the worst quality. In conclusion, the WS treatment performs best in regulating enzyme activity, balancing anti-oxidant capacity and browning control.

Catalase (CAT) and superoxide dismutase (SOD) are two key enzymes in the plant anti-oxidant defense system (Figure 4C,D). They function in a coordinated manner to scavenge reactive oxygen species (ROS) and play complementary roles in alleviating oxidative stress and delaying tissue senescence [44,45]. On day 0 of storage, the initial CAT and SOD activities of all treatment groups are 7.66 U/mL and 3721.33 U/L respectively. With the extension of storage time, the activities of both enzymes show a trend of first increasing and then decreasing. In the early stage of storage, fresh-cut injury induces the burst of reactive oxygen species, and at the same time activates the anti-oxidant response mediated by SOD and CAT: SOD rapidly scavenges excess O_2_^−^·, and CAT simultaneously scavenges H_2_O_2_ generated by the disproportionation reaction, and the activities of the two enzymes increase synergistically to resist oxidative stress [44]. On day 7 of storage, the SOD and CAT activities of all groups reach the peak: the SOD peaks of the CT group (4873.46 U/L) and WS group (4374.61 U/L) are the highest; in terms of CAT activity, the activities of the HO, WS and CT groups are all higher than that of the control group, indicating that these treatments effectively induce the activation of the anti-oxidant enzyme system. On day 14 of storage, the two enzyme activities of the HO and CT groups maintain advantages: their CAT activities are 9.12 U/mL and 9.18 U/mL respectively, and their SOD activities are significantly higher than that of the control group (4366.54 U/L), indicating that these two treatments effectively delay the decline of SOD and CAT activities, maintain the integrity of the ROS scavenging cascade reaction, thus continuously alleviating oxidative stress damage and delaying the senescence process [29]. Overall, both the HO and CT treatments can effectively activate and maintain the SOD-CAT combined anti-oxidant system in fresh-cut apples, and improve the tissue’s complete scavenging capacity for O_2_^−^· → H_2_O_2_ → H_2_O [45], among which the CT treatment performs most prominently in inducing the peak of SOD activity and maintaining the later combined scavenging capacity.

Phenylalanine ammonia-lyase (PAL) is the first key enzyme in the polyphenol synthesis pathway of fruits and vegetables [18]. As shown in Figure 4E, with the extension of storage time, the PAL enzyme activity of fresh-cut apples in all treatment groups shows a trend of rapid rise first and then tends to be stable. On day 0 of storage, the PAL activity of all groups is 40.56 U/L. After 7 days of storage, the PAL activity of all treatment groups increases significantly, indicating that mechanical injury induces the rapid activation of PAL, which is an important physiological response of plant tissues to initiate defense response and synthesize phenolic substances. On day 14 of storage, the PAL activity of all treatment groups is significantly higher than that of the control group (CK), among which the PAL activity of the CT treatment group is the highest, 14.19% higher than that of the CK group. The PAL activity does not continue to rise but tends to be stable in the late storage period, which may be because the accumulation of phenolic substances and their metabolites produces feedback inhibition on PAL activity, thus maintaining it at a relatively stable high level. These results show that pre-cutting high oxygen combined with ashwagandha extract treatment (CT) can significantly and continuously improve the PAL activity of fresh-cut apples. As a key enzyme in the phenylpropane metabolic pathway, the increase in PAL activity can promote the synthesis of stress-related substances such as phenols, flavonoids and lignin, thus improving the defense ability of apple tissues against cutting injury and reducing the risk of oxidative stress and browning caused by mechanical injury [46,47]. The CT treatment provides an enzymatic basis for the continuous synthesis of phenolic substances by maintaining high PAL activity, which is consistent with its excellent performance in total phenol content and anti-oxidant activity described above.

### 3.5. Changes in the Proteomic Profile of Fresh-Cut Apples Revealed by High-Throughput Proteomics

#### 3.5.1. Data Quality and Overall Sample Differences

A total of 85,599 peptides and 9900 proteins were identified in this proteomic analysis. The number of peptides is significantly higher than the number of proteins, indicating high protein coverage depth, which can provide support for subsequent differential expression and functional enrichment analysis. The coefficient of variation in protein quantification within each treatment group is low, indicating that the sample preparation and mass spectrometry detection processes are stable, and the obtained quantitative data are highly reliable.

**Figure 5:**
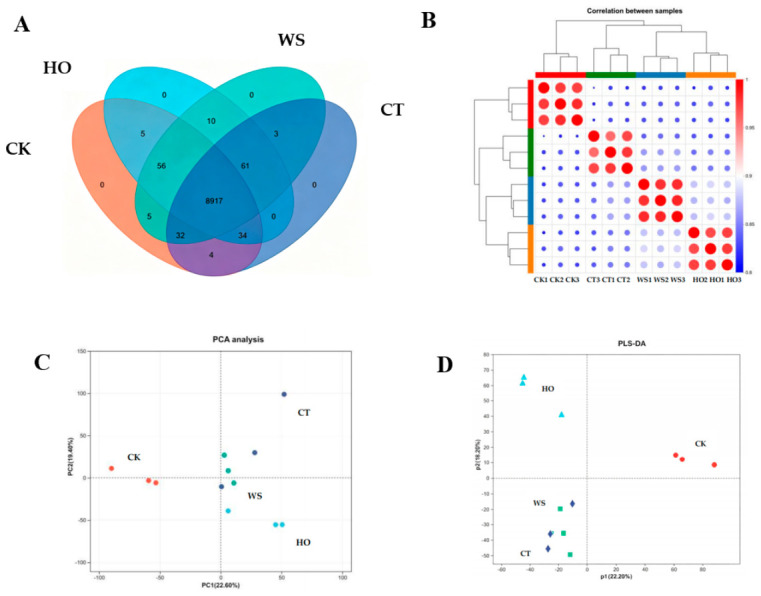
Protein Venn diagram (**A**), protein correlation heat map (**B**), protein PCA (**C**) and protein PLS-DA (**D**). Note: Figure 5A. Orange, blue, green, and purple lines indicate CK, HO, WS, and CT groups, respectively. Figure 5C. Red, blue, green, and purple dots indicate CK, HO, WS, and CT groups, respectively. Figure 5D. Red dots, blue triangles, green squares, and purple diamonds indicate CK, HO, WS, and CT groups, respectively.

The protein Venn diagram (Figure 5A) shows that the four groups have a total of 8917 common proteins, accounting for 97.70% of the total identified proteins, indicating that different treatments do not change the overall framework of protein detection, and the differences are mainly reflected in the redistribution of expression abundance of common proteins. The correlation between biological replicates of each treatment group is high: the correlation coefficient of the CK group is 0.976~0.983, that of the HO group is 0.972~0.978, that of the WS group is 0.981~0.985, and that of the CT group is 0.961~0.979.

Based on the types and responses of proteins in each treatment group, the similarity of the whole proteome among the four groups was investigated using Pearson correlation analysis (Figure 5B), PCA (Figure 5C), and PLS-DA (Figure 5D). The results of Pearson correlation analysis showed that the similarity among biological replicates within each group was high, with correlation coefficients all exceeding 0.95.

The PCA results (Figure 5B) show that the CK group samples are mainly distributed in the negative region of PC1, which is clearly separated from the other three treatment groups, indicating that the treatment factor is the primary driver of the differential protein expression in fresh-cut apples on day 7. The HO group is mainly distributed in the positive region of PC1 and the negative region of PC2, most of the CT group is located in the positive region of PC1, and the WS group partially overlapped with the CT group, primarily located in the positive regions of PC1 and PC2. The PLS-DA model exhibited R^2^X (cum) = 0.404, R^2^Y (cum) = 0.627, and Q^2^ = 0.226, with the Q^2^ intercept from 200 permutation tests being less than 0, indicating that the model was not overfitted. The PLS-DA results were generally consistent with those of the PCA, showing that the different treatment groups were largely well distinguished from one another. However, the samples in the WS and CT groups exhibited a high degree of clustering, indicating that the protein expression patterns between these two treatment groups were highly similar.

All identified proteins were annotated based on the Uniprot, GO, KEGG, EggNOG and Pfam databases, and corresponding annotation information was obtained from each database, mainly involving basic metabolism, energy conversion, post-translational regulation of proteins, intracellular transport, redox regulation and other processes (detailed annotation statistics provided in Supplementary Section S3.5.1).

#### 3.5.2. Differential Protein Analysis

**Figure 6:**
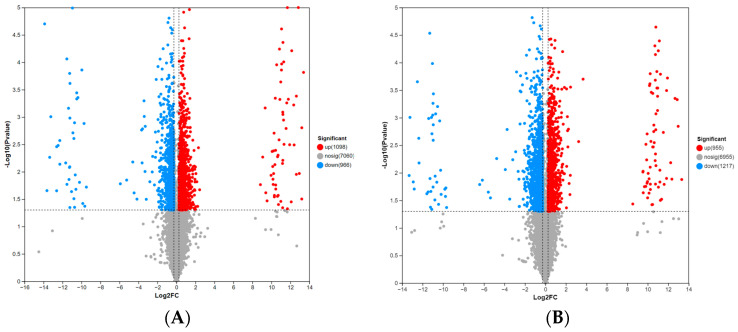

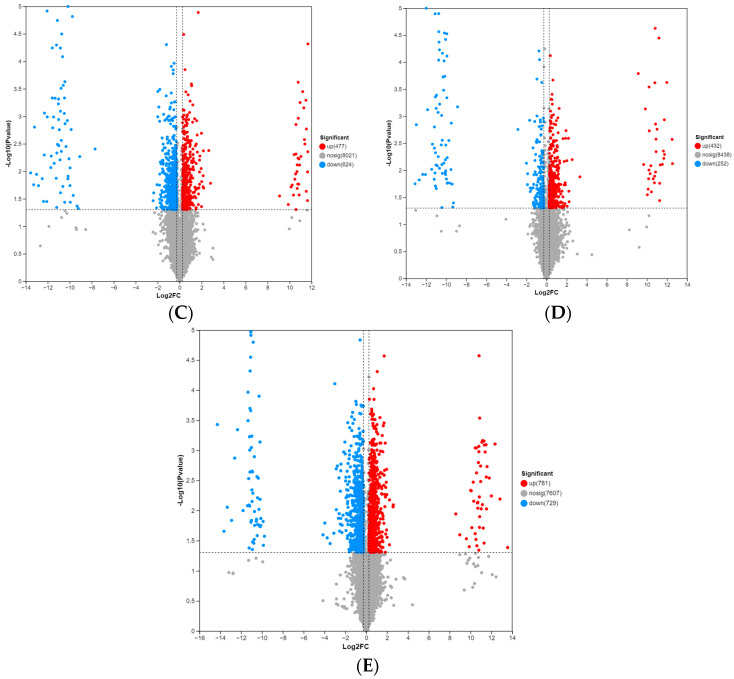
Volcano plots of the pairwise differential comparison of proteins between the HO group and CK group (**A**), the WS group and CK group (**B**), the CT group and HO group (**C**), the CT group and WS group (**D**), the CT group and CK group (**E**). Note: Each dot in the figure represents a specific protein: dots on the left represent down-regulated proteins, and dots on the right represent up-regulated proteins; the closer a dot is to the left and right ends and the higher its position is, the more significant the differential expression is.

As shown in Figure 6, differential protein analysis is carried out using the screening criteria of |log_2_FC| ≥ 1 and *p* < 0.05. The pairwise comparison results show that a total of 2064 differentially expressed proteins are screened in HO vs. CK, including 1098 (53.2%) up-regulated proteins and 966 (46.8%) down-regulated proteins; a total of 2172 differentially expressed proteins are screened in WS vs. CK, including 955 (44.0%) up-regulated proteins and 1217 (56.0%) down-regulated proteins; a total of 1510 differentially expressed proteins are screened in CT vs. CK, including 781 (51.7%) up-regulated proteins and 729 (48.3%) down-regulated proteins. Notably, relative to the individual treatments, the combined treatment group (CT) exhibited a reduction of approximately 25–30% in the number of DEPs compared with CK, suggesting that the CT treatment may attenuate proteomic fluctuation in fresh-cut apples during storage through the pre-activation and pre-regulation of cellular processes. A total of 1101 differentially expressed proteins are screened in CT vs. HO, including 477 up-regulated proteins and 624 down-regulated proteins; a total of 684 differentially expressed proteins are screened in CT vs. WS, including 432 up-regulated proteins and 252 down-regulated proteins. Of particular note, the relatively small number of down-regulated proteins between CT and WS indicates a modest proteomic disparity between these two groups, implying that the combined treatment and the ashwagandha-alone treatment may exert analogous functional profiles. Collectively, while both the WS and HO treatments elicited extensive proteomic reprogramming relative to CK, the CT treatment, despite integrating the effects of both interventions, demonstrated a more convergent and tightly regulated proteomic landscape.

Differential proteomic profiling revealed that the HO and WS treatments triggered distinct regulatory modules including cell wall, stress signaling versus membrane lipid, and anti-oxidant defense, respectively. The combined CT treatment, however, exhibited a more integrated pattern: it not only enhanced stress repair and phospholipid turnover relative to HO, but also restored certain signaling and repair components relative to WS. Moreover, CT uniquely modulated plastidial electron transport and transmembrane flux. Collectively, these findings indicate that the browning inhibitory effect of CT arises from a multi-tiered rebalancing of redox homeostasis, membrane dynamics, and protein quality control, rather than from simple additive effects of the two individual treatments. Detailed protein lists and fold changes are provided in Supplementary Section S3.5.2.

#### 3.5.3. GO Functional Enrichment Analysis

**Figure 7:**
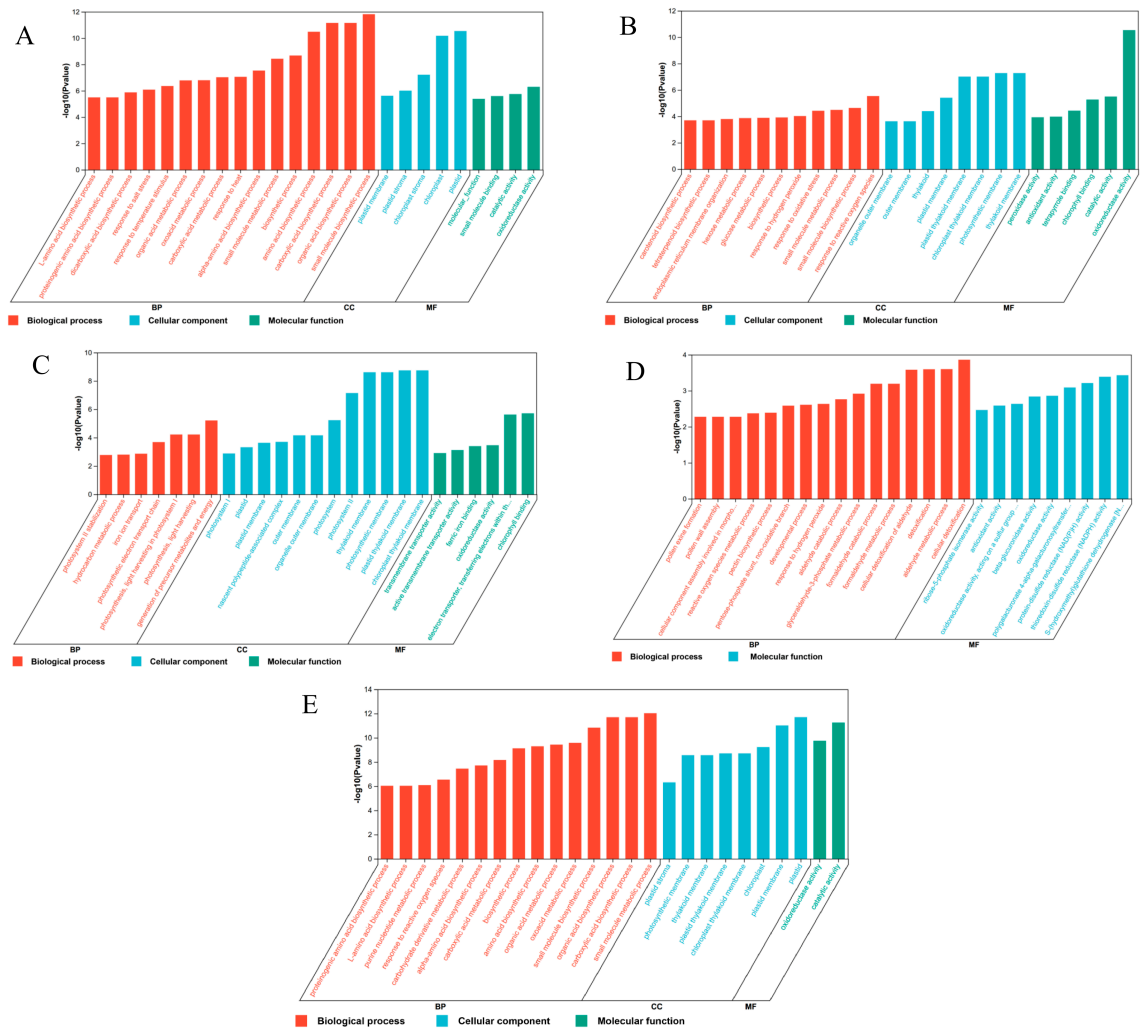
GO functional annotation of the differential proteins between the HO group and CK group (**A**), the WS group and CK group (**B**), the CT group and HO group (**C**), the CT group and WS group (**D**), the CT group and CK group (**E**). Note: *p*-adjust ≤ 0.5, only the top 20 enrichment results are shown.

GO functional enrichment analysis was performed to characterize the biological processes (BP), cellular components (CC), and molecular functions (MF) of DEPs across treatment groups (Figure 7). Compared with CK, the HO treatment primarily enriched BP terms related to biosynthesis, small molecule metabolism, and amino acid metabolism, with CC mainly involving plastids and chloroplasts, and MF dominated by catalytic and oxidoreductase activities (Figure 7A), suggesting that hyperoxia alters basal metabolism and plastidial redox activity. In contrast, WS vs. CK enrichment was centered on carotenoid biosynthesis, oxidative stress response, and ROS scavenging, with peroxidase and anti-oxidant activities as major MF terms (Figure 7B), consistent with the extract’s role in enhancing free radical elimination. For CT vs. HO (Figure 7C), enriched terms included carbohydrate metabolism, iron ion transport, and photosynthetic electron transport, indicating that CT further adjusts plastidial metabolism and electron flux beyond HO treatment. CT vs. WS (Figure 7D) showed enrichment in pectin biosynthesis, hydrogen peroxide response, and aldehyde catabolism, implying that CT may improve tissue quality via cell wall remodeling and active aldehyde detoxification. Finally, CT vs. CK (Figure 7E) exhibited broad enrichment in amino acid biosynthesis, purine nucleotide metabolism, ROS response, and organic acid metabolism, confirming that CT exerts systemic modulation of both basal metabolic networks and oxidative stress pathways. GO functional enrichment analysis of each comparison group is presented in Supplementary Section S3.5.3.

#### 3.5.4. KEGG Pathway Enrichment Analysis

**Figure 8:**
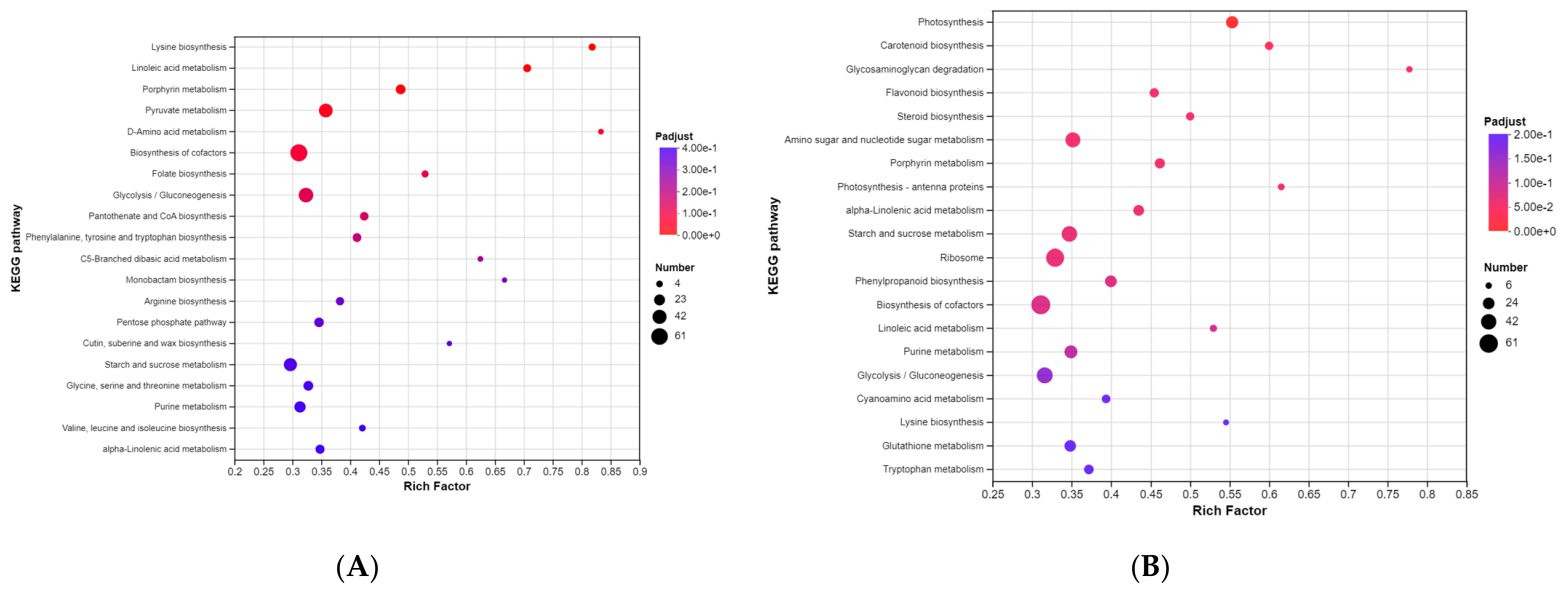

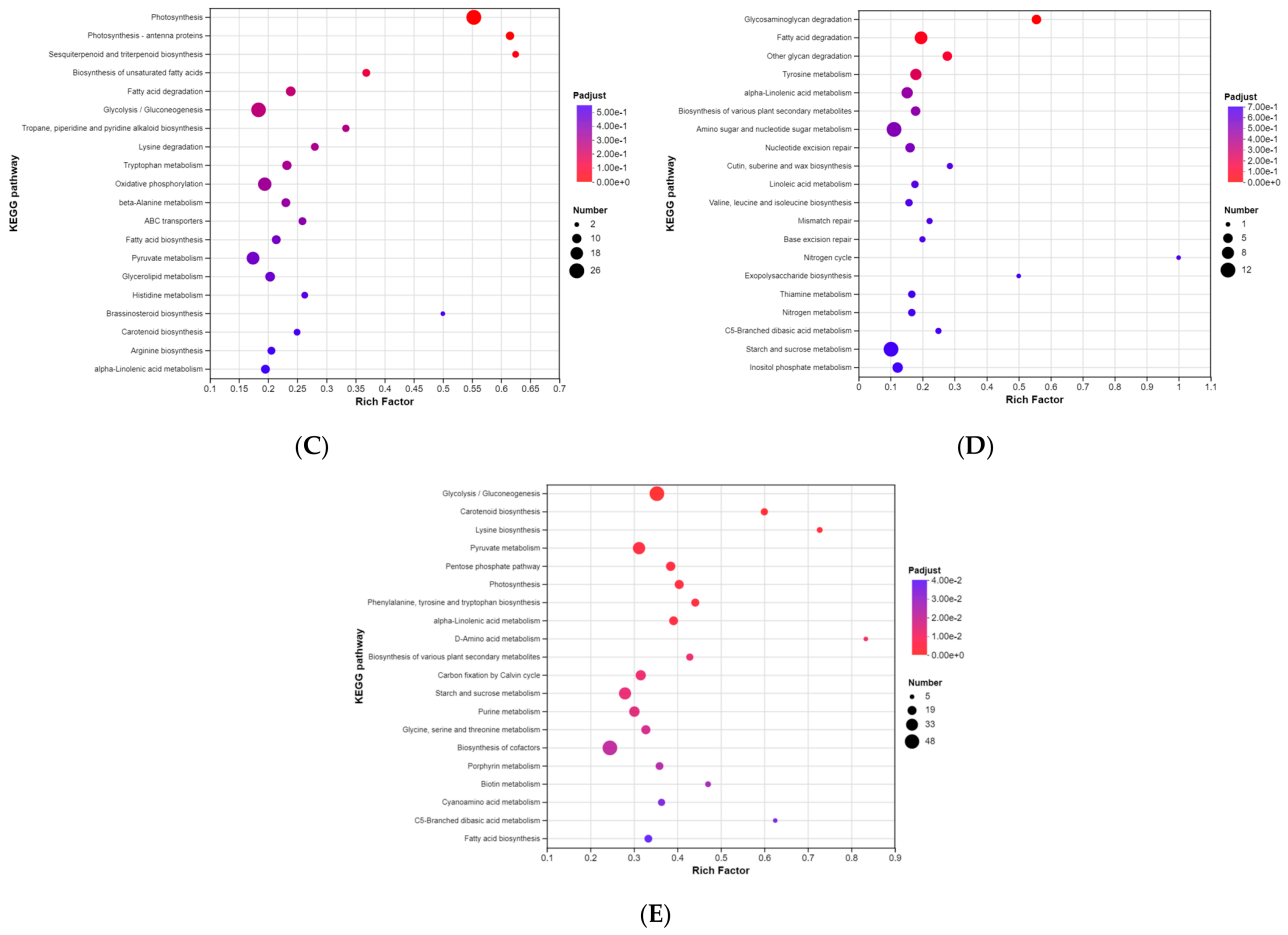
The KEGG enrichment pathway analysis of differential proteins between the HO group and CK group (**A**), the WS group and CK group (**B**), the CT group and HO group (**C**), the CT group and WS group (**D**), the CT group and CK group (**E**). Note: Rich factor represents the enrichment rate, which is calculated by dividing the number of proteins enriched in a certain pathway (protein number) by the total number of annotated proteins in this pathway (Background number). The higher the ratio, the higher the enrichment degree. The bubble size corresponds to the number of proteins enriched in the KEGG pathway, and the bubble color represents the Padjust value (corrected *p* value, *p*-adjust ≤ 1). Only the top 20 pathways with the highest enrichment degree are shown.

KEGG pathway enrichment analysis (Figure 8A–E) revealed that a set of core pathways were consistently and stably enriched across all comparison combinations, including biosynthesis of cofactors, glycolysis/gluconeogenesis, pyruvate metabolism, starch and sucrose metabolism, the pentose phosphate pathway, and α-linolenic acid metabolism. This highly conserved enrichment pattern suggests that, irrespective of high oxygen treatment, natural extract intervention, or their synergistic combination, the fundamental reprogramming of carbon flux distribution and energy metabolism pathways is universally induced in fresh-cut apples. Notably, the pentose phosphate pathway was persistently identified in each comparison, underscoring its potential prominence in the metabolic adaptation to post-cutting stress. Despite this commonality, distinct differences were observed among the treatments: in the HO vs. CK comparison, enrichment was primarily focused on carbon skeleton rearrangement and primary energy supply pathways (Figure 8A); in the WS vs. CK comparison, additional modules related to protein synthesis and anti-oxidant defense were superimposed onto the basal metabolic framework (Figure 8B); CT vs. HO further regulated energy conversion and lipid turnover (Figure 8C); CT vs. WS additionally affected cell wall metabolism, lipid degradation, and damage repair (Figure 8D); and CT vs. CK integrated nearly all core pathways, exhibiting the broadest metabolic coverage, with PPP and glutathione metabolism jointly providing NADPH and peroxide-scavenging capacity to maintain redox homeostasis and retard browning (Figure 8E). Detailed KEGG enrichment pathway analyses of DEPs in each comparison group are available in the Supplementary Section S3.5.4.

#### 3.5.5. Molecular Mechanism Diagram of Browning Delay in Fresh-Cut Apples

**Figure 9:**
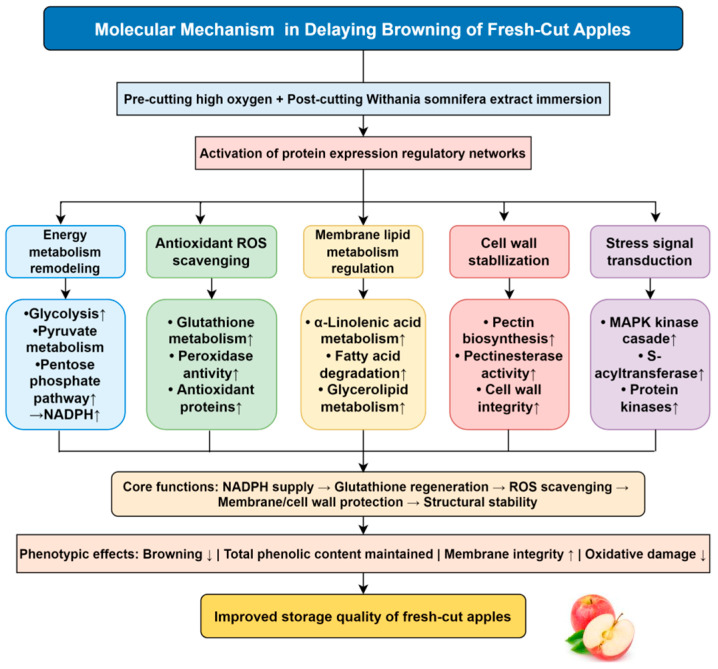
A schematic diagram of the molecular mechanism of the combined treatment (CT) delaying the browning of fresh-cut apples. Note: upward and downward arrows indicate increase and decrease, respectively.

To further clarify the molecular mechanism of the combined treatment (CT) delaying the browning of fresh-cut apples, a schematic model of its action mechanism is constructed based on the results of differential protein expression, GO annotation and KEGG pathway enrichment analysis (Figure 9). The results show that HO mainly activates proteins related to cell wall remodeling and stress signal transduction, but partially consumes the anti-oxidant buffer capacity. WS focuses on enhancing glutathione metabolism and ROS scavenging, showing a regulatory mode dominated by anti-oxidant defense. Notably, the combined treatment (CT) is not a simple superposition of the effects of HO and WS, but produces coordinated responses at multiple levels such as energy metabolism, membrane lipid turnover, cell wall metabolism, stress repair, ROS clearance, etc. The mechanism of CT delaying browning involves the integrated regulation of the following multiple pathways.

First, at the level of energy and reducing power remodeling, CT significantly up-regulates the expression levels of proteins related to glycolysis/gluconeogenesis, pyruvate metabolism, and the pentose phosphate pathway; increases the supply of NADPH; and provides reducing power support for glutathione regeneration. Second, at the level of redox homeostasis maintenance, CT activates pathways related to glutathione metabolism and ROS response, and increases the expression levels of peroxidase and anti-oxidant proteins, thereby effectively alleviating the oxidative stress caused by cutting and storage. Third, at the level of membrane lipid metabolism regulation, CT promotes α-linolenic acid metabolism, fatty acid degradation and glycerolipid metabolism, reducing membrane lipid peroxidation damage and maintaining membrane integrity, thereby limiting enzyme–substrate contact. Fourth, at the level of cell wall stability regulation, CT up-regulates the proteins related to pectin biosynthesis and pectinesterase activity, reinforcing cell wall structure to restrict phenolic substrate release and inhibit enzymatic browning. Fifth, at the level of stress signal transduction, CT activates the MAPK kinase cascade and the expression of S-acyltransferase and a variety of protein kinases. These signal molecules play a key role in sensing oxidative stress and regulating downstream defense responses, helping cells quickly adapt to the adverse environment during storage.

The concurrent regulation of these five pathways finally converges into a core functional chain: “NADPH supply → Glutathione regeneration → ROS clearance → Membrane/cell wall protection → Structural stability”. Each link of this chain supports each other and progresses layer by layer: NADPH, as a source of reducing power, drives the glutathione cycle, and glutathione can reduce membrane lipid peroxidation and cell wall degradation after clearing ROS, thereby maintaining the overall stability of cell structure. With the execution of these core functions, CT shows a significant anti-browning effect at the phenotypic level, which is specifically reflected in the reduced browning index, stable total phenol content, improved membrane integrity, and reduced oxidative damage, and finally achieves the overall improvement of the storage quality of fresh-cut apples. In summary, CT systematically delays the browning process of fresh-cut apples through the hierarchical regulatory pathway of “upstream treatment → protein network activation → integrated multi-pathway regulation → core function execution → phenotypic effect”. Subsequent targeted verification of key proteins (such as by PRM or Western blot) is also needed to further confirm the reliability of these proteomics results.

## 4. Conclusions

This study systematically evaluates the effects of pre-cutting high oxygen treatment (90% O_2_, 3 h) combined with post-cutting *Withania somnifera* extract immersion on the storage quality and anti-browning mechanism of fresh-cut apples. The results show that after 14 days of storage, the combined treatment (CT) group has the lowest browning index and the highest whiteness index, and the color protection effect is significantly better than that of the two single treatments. The combined treatment increases the total phenol content and DPPH free radical scavenging rate, thereby enhancing the anti-oxidant capacity. Although the contents of superoxide anion and H_2_O_2_ increase in the late storage period, the combined treatment group maintains higher activities of SOD, CAT and POD, which alleviates oxidative damage to a certain extent, which can also be confirmed by the lower malondialdehyde content and better membrane integrity. In addition, the combined treatment increases the activity of phenylalanine ammonia-lyase to promote the synthesis of phenolic substances, but also induces higher PPO activity at the same time, indicating that the treatment intensity needs to be further optimized to avoid potential browning risks. Proteomics analysis shows that the CT treatment is not a simple superposition of the effects of the two single treatments, but systematically delays browning from multiple levels such as improving energy supply, reducing power generation, redox homeostasis maintenance, structural integrity protection, etc., through integrated regulation of multiple pathways such as glycolysis, pentose phosphate pathway, glutathione metabolism, fatty acid metabolism, cell wall metabolism, etc. In summary, pre-cutting high oxygen treatment combined with post-cutting *Withania somnifera* extract treatment is an effective anti-browning strategy for fresh-cut apples, and its mechanism involves reactive oxygen species clearance, membrane lipid protection, phenolic metabolism regulation and coordinated multi-pathway responses, which provides new ideas and scientific basis for the development of green and efficient fresh-cut fruit and vegetable preservation technology. However, this study still has certain limitations: the mechanism interpretation is currently mainly based on correlation analysis, and direct verification is still needed; the phenomena of increased PPO activity and late ROS accumulation indicate that treatment parameters such as oxygen concentration, treatment duration, extract dosage, etc., need to be further optimized; in addition, the results of this study are only for a single variety and a single storage condition, and their universality remains to be confirmed. Therefore, although this strategy has good application prospects, practical application still needs to be cautious, and further research under a wider range of conditions is needed to verify relevant mechanisms and optimize treatment schemes.

## Data Availability

The original contributions presented in this study are included in the article/Supplementary Materials. Further inquiries can be directed to the corresponding author.

## Supplementary Materials

The following supporting information can be downloaded at https://www.mdpi.com/article/10.3390/foods15152673/s1.
