# Supplementary material for: Combined Anti-Browning Mechanism of Pre-Cutting High Oxygen Treatment and Post-Cutting Withania somnifera Extract Treatment on Fresh-Cut Apples
Source: Foods. 2026 Jul 29;15(15):2673. doi: 10.3390/foods15152673 (PMC13465051; doi:10.3390/foods15152673)
Supplement: Supplementary file 1 [file foods-15-02673-s001.zip › foods-4408020-supplementary.pdf]

S2.6. High-throughput Proteomics

S2.6.1. Preparation of Protein Solution

Transfer part or all of the frozen samples to a grinding tube, add an appropriate amount of BPP solution. Grind the samples with a grinder for 3 times, 180 s each time. Centrifuge at 8 ℃ and 14000 g for 15 min then collect the supernatant, add equal volume of Tris-saturated phenol, and vortex for 5~10 s. Centrifuge at 8 ℃ and 14000 g for 15 min then collect the phenol phase, add equal volume of BPP solution, and vortex for 5~10 s. Centrifuge the mixture again at 8 ℃ and 14000 g for 15 min, and collect the phenol phase. Add 4 times volume of pre-cooled ammonium acetate methanol solution, and precipitate protein overnight at -20 ℃. The next day, centrifuge at 8 ℃ and 14000 g for 15 min then discard the supernatant, and wash the precipitate twice with 90% pre-cooled acetone. Dissolve the precipitate in protein lysis buffer (8 M urea + 1% SDS, containing protease inhibitor cocktail). Perform non-contact low-temperature ultrasonic treatment on the sample for 30 min, then centrifuge at 8 ℃ and 14000 g for 15 min, and collect the protein supernatant. BCA assay, SDS-PAGE or PA method is used to evaluate protein quality.

S2.6.2. Preparation of Polypeptide Samples

According to protein concentration, take 30~50 μg of protein from each sample, add an appropriate amount of lysis buffer, then add TEAB to make its final concentration 100 mM. Add TCEP to make its final concentration 10 mM, place the mixture in a shaker at 37 ℃ for incubation for 60 min. Add IAM (iodoacetamide) to make its final concentration 40 mM, and incubate at room temperature in the dark for 40 min. Then add 800 μL of acetone solution, and precipitate protein at -20 ℃ for 2 h. Centrifuge at 12000 g for 15 min then discard the supernatant, and wash the precipitate with 800 μL of 90% acetone salt solution. Centrifuge again at 12000 g for 15 min then discard the supernatant, and place the precipitate in a fume hood to air dry for 5 min. Add Trypsin according to the enzyme-protein ratio of 1:40 (w/w), and perform overnight enzymolysis on the sample under shaking condition at 37 ℃.

S2.6.3. Polypeptide Desalination

Add 200 μL of acetonitrile to activate the column once, add 200 μL of 0.1% TFA to equilibrate the column once. Load the sample with pH < 3 onto the column twice. Add 800 μL of 0.1% TFA to wash the column once, then add 200 μL of pure water to wash once. Add 100 μL of 70% acetonitrile for elution once.

S2.6.4. Polypeptide Quantification

Polypeptide quantification is performed by ultraviolet spectrophotometry using NANO DROP ONE (Thermo Scientific).

S2.6.5. Mass Spectrometry Detection

S2.6.5.1. Liquid Chromatography Method

Take equal amount of polypeptides dissolved in mass spectrometry loading buffer for DIA detection and analysis. Data acquisition software: Thermo Xcalibur 4.7 (Thermo, USA). Analytical column information: self-made chromatographic column (15 cm × 100 μm, 1.7 μm). Chromatograph: Vanquish Neo (Thermo, USA). Mass spectrometer: Astral (Thermo, USA). Chromatographic separation duration: 8 min. Mobile phase A: 2% acetonitrile solution containing 0.1% formic acid; Mobile phase B: 80% acetonitrile solution containing 0.1% formic acid.

Table S1. Liquid phase gradient.

Time (min)	B (%)	Flow rate (μL/min)
0	8	1
1	17	1
5.5	55	0.4

7	99	0.7
8	99	1

S2.6.5.2. Mass Spectrometry

Samples separated by nano-flow high performance liquid chromatography are subjected to DIA mass spectrometry analysis using an ASTRAL mass spectrometer. The detection mode is positive ion mode, and the ion source voltage is set to 2.2 kV. MS and MS/MS are detected and analyzed by the mass spectrometer. Data acquisition mode: DIA. MS1 scanning range: 380-980 m/z; MS2 scanning range: 150-2000 m/z.

S3.5.1. Functional annotation of all identified proteins

All identified proteins were annotated based on Uniprot, GO, KEGG, EggNOG and Pfam databases, and corresponding annotation information was obtained from each database, mainly involving basic metabolism, energy conversion, post-translational regulation of proteins, intracellular transport, redox regulation and other processes (detailed annotation statistics provided in Supplementary Figures S1–S4).

GO functional annotation (Figure S1(A)) shows that the annotated entries were classified into three main categories: Biological Process (BP), Molecular Function (MF), and Cellular Component (CC). At the biological process level, the identified proteins are widely involved in metabolic processes, localization establishment, biological process regulation, cellular component organization or biogenesis, cellular localization, stress response and other biological processes. These findings indicate that the identified proteins are extensively involved in key biological functions such as substance metabolism, regulation of intracellular localization, and stress responsiveness. In the CC category, the differentially expressed proteins were mainly localized to components such as membrane, organelle, cytoplasm, and cytosol, suggesting that their functional execution is closely associated with the membrane system, organelles, and the cytoplasmic matrix. In the cellular component category, the annotated proteins are mainly distributed in membrane structures, organelles, cytoplasm and cytosol, and are also abundantly distributed in components such as catalytic complexes, ribonucleoprotein complexes, and membrane protein complexes, indicating that these proteins are mainly located in membrane systems, organelles and cytoplasm-related structures. In the molecular function category, proteins are mainly enriched in small molecule binding, organic cyclic compound binding, transferase activity, hydrolase activity. In addition, categories such as carbohydrate derivative binding, oxidoreductase activity, protein binding, and transmembrane transporter activity also account for a relatively high proportion, indicating that the identified proteins are mainly related to molecular binding, enzyme catalysis and transmembrane transport functions.

The results of KEGG pathway annotation are shown in Figure S1 (B). The annotated entries were classified into five main categories: Cellular Processes, Environmental Information Processing, Genetic Information Processing, Metabolism, and Organismal Systems. Among these, the Metabolism category accounted for the highest proportion, mainly comprising Carbohydrate metabolism (623 entries), Amino acid metabolism (351), Lipid metabolism (282), Energy metabolism (275), and Metabolism of cofactors and vitamins (263). In addition, pathways in the Genetic Information Processing category, including Folding, sorting and degradation (473) and Translation (288), as well as Transport and catabolism (316) in the Cellular Processes category, also occupied relatively

large proportions, suggesting that the apple tissues after fresh-cut processing remained in a relatively active state of metabolism and protein turnover.

In the EggNOG classification (Figure S1 (C)), categories such as post-translational modification, protein turnover, chaperones, as well as intracellular transport, secretion and vesicular transport account for a relatively high proportion, indicating that under storage stress, protein homeostasis and intracellular transport processes are relatively active. In addition, the Pfam annotation results (Figure S1 (D)) showed that proteins containing domains such as the PPR repeat family (PPR_2), leucine-rich repeat (LRR_8), protein kinase domain (Pkinase), and WD40 domain were relatively abundant.

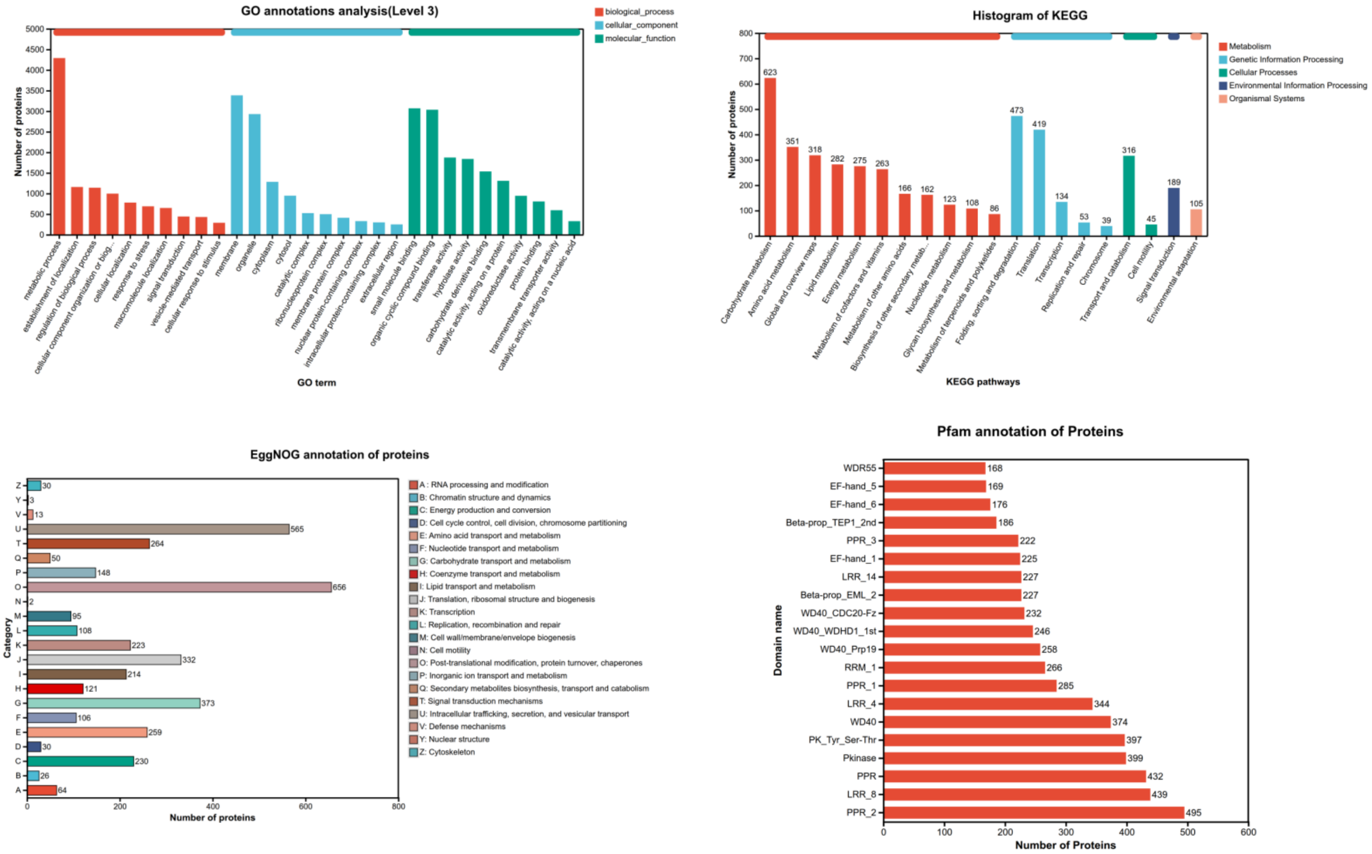

Figure S1. GO functional annotation of total proteins(A), KEGG functional annotation of total proteins(B), EggNOG functional annotation of total proteins(C), and Pfam Annotation of Total Proteins(D)

S3.5.2. Differential Protein Analysis

As shown in Table S2, in HO vs CK, proteins including pectinesterase, Fe2OG dioxygenase domain-containing protein, mitogen-activated protein kinase kinase and methylcoclaurine hydroxylase are up-regulated, while proteins including glutathione transferase, SHSP domain-containing protein and serine/threonine protein phosphatase are down-regulated. This indicates that hyperoxia treatment activates cell wall remodeling and stress signal transduction on the one hand, but may be accompanied by the consumption of part of the antioxidant buffering capacity on the other hand.

Table S2. Differential Proteins Between HO Group and CK Group

Accession Number	Protein Name	FC	P-value	Regulation Direction
Q6V8T8	Methylcoclaurine hydroxylase	10715.15	0.000143	Up-regulated
A0A498K4L1	Purple acid phosphatase N-terminal domain-containing protein	9425.056	0.001562	Up-regulated
A0A498JGA5	AB hydrolase-1 domain-containing protein	9400.91	0.03147	Up-regulated
A0A498I0I0	Rhodanese domain-containing protein	7869.432	0.01075	Up-regulated
A0A498KLN7	Pectinesterase	7323.414	1.54E-08	Up-regulated
A0A498K191	Ribosome maturation factor RimP N-terminal domain-containing protein	6341.862	0.000406	Up-regulated
A0A498HUE6	Fe2OG dioxygenase domain-containing protein	6336.904	0.01115	Up-regulated
A0A498JJM7	S-acyltransferase	5386.744	0.003228	Up-regulated
A0A498KP21	Rhodopsin	5229.151	0.000593	Up-regulated
A0A498IFH4	CBM20 domain-containing protein	4518.858	5.16E-05	Up-regulated
A0A498INB7	Fe2OG dioxygenase domain-containing protein	4362.026	0.03547	Up-regulated
A0A498ISY5	Fe2OG dioxygenase domain-containing protein	3886.761	0.003201	Up-regulated
A0A498KST0	HMG box domain-containing protein	3535.346	0.000464	Up-regulated
Q8LKE6	Putative disease resistance gene analog NBS-LRR	3308.886	0.04744	Up-regulated
A0A498KLA5	mitogen-activated protein kinase kinase	3297.991	4.32E-08	Up-regulated
A0A498HPF6	Uncharacterized protein	6.62E-05	9.91E-06	Down-regulated
A0A498IV99	Uncharacterized protein	7.84E-05	0.02212	Down-regulated
A0A498HZB7	EF-hand domain-containing protein	9.58E-05	0.005441	Down-regulated
A0A498KSW2	Protein kinase domain-containing protein	0.000104	0.000978	Down-regulated
A0A498J8D7	Phospholipase/carboxylesterase/thioesterase domain-containing protein	0.000159	0.02225	Down-regulated
A0A498HZ14	EF-hand domain-containing protein	0.000159	0.003477	Down-regulated
A0A498JRJ7	glutathione transferase	0.000177	0.003305	Down-regulated
A0A498K2T5	Uncharacterized protein	0.0002	0.002681	Down-regulated
A0A498JI05	Leucine-rich repeat-containing N-terminal plant-type domain-containing protein	0.000201	0.007271	Down-regulated
A0A498HLL6	Serine/threonine-protein phosphatase	0.000309	0.006888	Down-regulated
A0A498K4H0	Uncharacterized protein	0.000335	7.69E-05	Down-regulated
A0A498HJD9	Ninja-family protein	0.000338	0.01698	Down-regulated
A0A498HRT6	Protein kinase domain-containing protein	0.000379	0.000682	Down-regulated
A0A498IPX2	SHSP domain-containing protein	0.000393	0.01384	Down-regulated
A0A498ICT6	Uncharacterized protein	0.000406	0.008319	Down-regulated

Note: Based on FC values, the list only displays the top 15 up-regulated and top 15 down-regulated differential proteins, $p < 0.05$.

Table S3 shows that compared with CK, the expression of proteins such as S-acyltransferase, putative disease resistance gene homolog NBS-LRR, pectinesterase, lipoxygenase, Fe2OG dioxygenase in WS is up-regulated, while the expression of some protein kinases, DNA ligase and GH18 domain-containing protein is down-regulated. This indicates that the extract treatment mainly affects processes related to membrane lipid metabolism, stress defense and redox. Subsequent enrichment analysis obtained annotations such as "response to reactive oxygen species" and "glutathione metabolism", which further shows that WS treatment is more inclined to the regulation mode centered on antioxidant defense.

Table S3. Differential proteins between WS group and CK group

Accession	Protein Name	FC	P value	Regulate
A0A498JGA5	AB hydrolase-1 domain-containing protein	10241.76	0.01322	Up-regulated
A0A498K4L1	Purple acid phosphatase N-terminal domain-containing protein	8040.587	0.001423	Up-regulated
A0A498I0I0	Rhodanese domain-containing protein	7540.475	0.00046	Up-regulated
A0A498JJM7	S-acyltransferase	6497.112	0.000437	Up-regulated
Q8LKE6	Putative disease resistance gene analog NBS-LRR	5254.551	0.01302	Up-regulated
A0A498KST0	HMG box domain-containing protein	4834.879	0.006549	Up-regulated
A0A498IFH4	CBM20 domain-containing protein	3896.518	0.000181	Up-regulated
A0A498JJL9	Uncharacterized protein	3666.756	0.000312	Up-regulated
A0A498IKU2	Zinc finger PHD-type domain-containing protein	3656.402	0.01263	Up-regulated
A0A498KLN7	Pectinesterase	3113.715	0.01619	Up-regulated
A0A498JF26	Lipoxygenase	2944.149	0.001838	Up-regulated
A0A498ISY5	Fe2OG dioxygenase domain-containing protein	2620.007	0.03015	Up-regulated
A0A498KGT2	Protein argonaute 5	2422.637	0.000152	Up-regulated
A0A498HQ88	Nucleolar GTP-binding protein 1	2411.58	0.03157	Up-regulated
A0A498HH95	Protein COFACTOR ASSEMBLY OF COMPLEX C SUBUNIT B CCB1, chloroplastic	2332.98	0.01468	Up-regulated
A0A498J7S1	EF-hand domain-containing protein	9.82E-05	0.01119	Down-regulated
A0A498KSW2	Protein kinase domain-containing protein	0.000104	0.000978	Down-regulated
A0A498IJW9	Uncharacterized protein	0.00013	0.01462	Down-regulated
A0A498IAL9	Fiber protein Fb34	0.000139	0.01968	Down-regulated
A0A498KFB0	Josephin-like protein	0.000172	0.000213	Down-regulated
A0A498K7F5	Uncharacterized protein	0.000189	0.002352	Down-regulated
A0A498IQ44	DNA ligase	0.000192	0.006613	Down-regulated
A0A498I010	Large ribosomal subunit protein uL22m	0.000325	0.02398	Down-regulated
A0A498KAJ0	GH18 domain-containing protein	0.000355	0.02225	Down-regulated
A0A498IKG1	non-specific serine/threonine protein kinase	0.000369	0.001023	Down-regulated
A0A498KIP6	Uncharacterized protein	0.000396	1.93E-05	Down-regulated
A0A498HT66	CHCH domain-containing protein	0.000404	0.0127	Down-regulated
A0A498IS77	VQ domain-containing protein	0.000417	0.04208	Down-regulated
A0A0M4F9N7	Histidine-containing phosphotransfer protein	0.000429	0.02177	Down-regulated
E4Z8N5	Putative RNA-directed DNA polymerase (Reverse transcriptase)	0.000446	0.00098	Down-regulated

Note: Based on the FC value, the list only displays the top 15 up-regulated differential proteins and the top 15 down-regulated differential proteins, $p < 0.05$.

Table S4 and Table S5 show that compared with HO and WS, CT also has additional expression remodeling conditions. Compared with HO, CT up-regulates proteins such as SHSP domain-containing protein, serine/threonine-protein phosphatase, phospholipase/carboxylesterase/thioesterase domain-containing protein, delta-1-pyrroline-5-carboxylate synthase, etc. Compared with WS, proteins up-regulated by CT include DNA ligase, histidine-containing phosphotransfer protein, non-specific serine/threonine protein kinase, and CHCH domain-containing protein. The above

changes indicate that the combined treatment does not simply improve antioxidant capacity, but builds a more suitable intracellular environment for delaying browning by simultaneously regulating membrane lipid turnover, stress repair, signal transduction and protein homeostasis.

Table S4. Differential proteins between CT group and HO group

Accession Number	Protein Name	FC	P value	Regulation Direction
A0A498HRT6	Protein kinase domain-containing protein	3259.444	3.83E-05	Up-regulated
A0A498HJD9	Ninja-family protein	3235.624	0.004402	Up-regulated
A0A498J8D7	Phospholipase/carboxylesterase/thioesterase domain-containing protein	3184.291	0.01024	Up-regulated
A0A498HMG2	Uncharacterized protein	3171.798	0.03407	Up-regulated
A0A498IQW4	hydroxymethylglutaryl-CoA lyase	2958.296	0.001691	Up-regulated
A0A498HLL6	Serine/threonine-protein phosphatase	2935.488	0.02312	Up-regulated
A0A498HYP9	PCI domain-containing protein	2872.284	0.000501	Up-regulated
A0A498IPX2	SHSP domain-containing protein	2688.415	0.003177	Up-regulated
A0A498JXU1	non-specific serine/threonine protein kinase	2615.166	0.00264	Up-regulated
A0A498IEA2	Delta-1-pyrroline-5-carboxylate synthase	2518.944	0.000691	Up-regulated
A0A498JI05	Leucine-rich repeat-containing N-terminal plant-type domain-containing protein	2344.468	0.000345	Up-regulated
A0A498K2T5	Uncharacterized protein	2101.636	0.005356	Up-regulated
A0A498K4H0	Uncharacterized protein	1987.91	0.000551	Up-regulated
A0A498KEQ9	DM2 domain-containing protein	1930.272	0.00771	Up-regulated
A0A498HPF6	Uncharacterized protein	1878.579	0.02693	Up-regulated
A0A498HA59	Potassium channel domain-containing protein	8.31E-05	0.01069	Down-regulated
A0A498JQP0	FKBP12-interacting protein of 37 kDa	0.0001	0.01756	Down-regulated
A0A498K4L1	Purple acid phosphatase N-terminal domain-containing protein	0.000106	0.001562	Down-regulated
A0A498J8L6	Plastocyanin	0.00012	0.01135	Down-regulated
A0A498JT63	Hydroxyproline-rich glycoprotein family protein	0.000136	0.01821	Down-regulated
A0A498IFH1	WEB family protein	0.000173	0.01358	Down-regulated

A0A498J113	Major facilitator superfamily (MFS) profile domain-containing protein	0.000187	0.03526	Down-regulated
A0A223A9V4	Cytochrome b6-f complex subunit 4	0.000196	0.005031	Down-regulated
A0A498IAA6	Uncharacterized protein	0.000199	0.000864	Down-regulated
A0A498INB7	Fe2OG dioxygenase domain-containing protein	0.000229	0.03547	Down-regulated
A0A498ING6	Protein DETOXIFICATION	0.000234	2.19E-06	Down-regulated
A0A498K8T7	Large ribosomal subunit protein uL22m	0.000236	0.001014	Down-regulated
A0A498KMA3	Major facilitator superfamily (MFS) profile domain-containing protein	0.000296	0.008552	Down-regulated
A0A498HQH8	AP2/ERF domain-containing protein	0.000304	0.005277	Down-regulated
A0A498II17	RRM domain-containing protein	0.000325	0.000454	Down-regulated

Note: Based on FC values, only the top 15 up-regulated differential proteins and the top 15 down-regulated differential proteins are displayed in the list, $p < 0.05$.

Table S5. Differential proteins between CT group and WS group.

Accession Number	Protein Name	FC	P value	Regulation Direction
A0A498KFB0	Josephin-like protein	5969.935	0.007532	Up-regulated
A0A498IQ44	DNA ligase	5815.157	0.002673	Up-regulated
A0A498IKG1	non-specific serine/threonine protein kinase	4011.249	0.000228	Up-regulated
A0A0M4F9N7	Histidine-containing phosphotransfer protein	3452.508	0.005087	Up-regulated
A0A498I010	Large ribosomal subunit protein uL22m	3395.244	0.001153	Up-regulated
A0A498KIP6	Uncharacterized protein	3272.042	0.006017	Up-regulated
A0A498HT66	CHCH domain-containing protein	3183.186	0.004399	Up-regulated
A0A498K7F5	Uncharacterized protein	2951.767	0.007768	Up-regulated
A0A498HZ49	Nuclear pore complex protein NUP1	2575.35	0.00795	Up-regulated
E4Z8N5	Putative RNA-directed DNA polymerase (Reverse transcriptase)	2465.409	0.01738	Up-regulated
A0A498IAL9	Fiber protein Fb34	2458.264	0.03632	Up-regulated
A0A498IBF1	Remorin C-terminal domain-containing protein	2343.762	2.59E-05	Up-regulated
A0A498HXA2	RING-type E3 ubiquitin transferase	2058.011	0.00173	Up-regulated
A0A498KAJ0	GH18 domain-containing protein	1926.732	0.004519	Up-regulated
A0A498KD81	Rab3GAP catalytic subunit conserved domain-containing protein	1919.507	0.002644	Up-regulated
A0A498JQP0	FKBP12-interacting protein of 37 kDa	0.000114	0.01777	Down-regulated
A0A498K4L1	Purple acid phosphatase N-terminal domain-containing protein	0.000124	0.001423	Down-regulated
A0A498J8L6	Plastocyanin	0.000152	0.01506	Down-regulated
A0A498JPU4	TIR domain-containing protein	0.000193	0.01189	Down-regulated

A0A498J113	Major facilitator superfamily (MFS) profile domain-containing protein	0.000235	0.01199	Down-regulated
A0A498IV99	Uncharacterized protein	0.000246	6.19E-08	Down-regulated
A0A498JT63	Hydroxyproline-rich glycoprotein family protein	0.000272	0.000747	Down-regulated
A0A498I5N9	S-adenosylmethionine-dependent methyltransferase	0.000359	0.009591	Down-regulated
A0A498JMD0	Uncharacterized protein	0.000379	0.003283	Down-regulated
A0A498HH95	Protein COFACTOR ASSEMBLY OF COMPLEX C SUBUNIT B CCB1, chloroplastic	0.000429	0.01468	Down-regulated
A0A498K8T7	Large ribosomal subunit protein uL22m	0.000435	0.003879	Down-regulated
A0A498IFH1	WEB family protein	0.000435	0.001568	Down-regulated
A0A498HCL7	non-specific serine/threonine protein kinase	0.000451	0.01118	Down-regulated
A0AU75	Mal d 1.03C02	0.000453	2.75E-06	Down-regulated
A0A498JB74	Nuclear pore complex protein NUP1	0.000465	0.000429	Down-regulated

Note: Based on the FC value, the list only displays the top 15 up-regulated differential proteins and top 15 down-regulated differential proteins in the ranking, $p < 0.05$.

As shown in Table S6, compared with the CK group, proteins including AB hydrolase-1 domain-containing protein, rhodanese domain-containing protein, S-acyltransferase, CBM20 domain-containing protein, Fe2OG dioxygenase domain-containing protein, lipoxygenase, etc. in the CT group are significantly up-regulated, while proteins such as plastocyanin, cytochrome b6-f complex subunit 4, major facilitator superfamily (MFS) profile domain-containing protein, etc. are down-regulated. This shows that the combined treatment will not only affect redox homeostasis, but also be accompanied by re-regulation of plastid-related electron transfer and transmembrane transport.

Table S6. Differential proteins between CT group and CK group

Accession Number	Protein Name	FC	P value	Regulation Direction
A0A498JGA5	AB hydrolase-1 domain-containing protein	12100.74	0.0413	Upregulated
A0A498I0I0	Rhodanese domain-containing protein	7296.173	0.006444	Upregulated
A0A498JJM7	S-acyltransferase	5250.639	0.000777	Upregulated
A0A498IFH4	CBM20 domain-containing protein	4119.159	0.005719	Upregulated
Q8LKE6	Putative disease resistance gene analog NBS-LRR	3580.77	0.002877	Upregulated
A0A498HUE6	Fe2OG dioxygenase domain-containing protein	3081.95	0.001855	Upregulated
A0A498HQ88	Nucleolar GTP-binding protein 1	2977.076	0.009399	Upregulated
A0A498JLK9	AB hydrolase-1 domain-containing protein	2928.377	0.002769	Upregulated
Q6V8T8	Methylcoclaurine hydroxylase	2886.521	0.000797	Upregulated
A0A498JF26	Lipoxygenase	2719.222	0.001056	Upregulated
A0A498I165	Nuclear speckle splicing regulatory protein 1 N-terminal domain-containing protein	2584.569	0.000809	Upregulated
A0A498HHF4	Uncharacterized protein	2535.926	0.03489	Upregulated
A0A498IEA2	Delta-1-pyrroline-5-carboxylate synthase	2518.944	0.000691	Upregulated
A0A498JUR8	UBC core domain-containing protein	2390.319	0.01963	Upregulated
A0A498KP21	Rhodopsin	2289.92	0.000679	Upregulated

A0A498J8L6	Plastocyanin	5.03E-05	0.000362	Downregulated
A0A498IV99	Uncharacterized protein	7.84E-05	0.02212	Downregulated
A0A498J113	Major facilitator superfamily (MFS) profile domain-containing protein	9.72E-05	0.008824	Downregulated
A0A498IJW9	Uncharacterized protein	0.00013	0.01462	Downregulated
A0A498JPU4	TIR domain-containing protein	0.00016	0.001337	Downregulated
A0A223A9V4	Cytochrome b6-f complex subunit 4	0.000193	0.000444	Downregulated
A0A498K8T7	Large ribosomal subunit protein uL22m	0.000279	0.01002	Downregulated
A0A498HFV4	Seed biotin-containing protein SBP65-like	0.000375	0.008327	Downregulated
A0A498JYP3	Fe2OG dioxygenase domain-containing protein	0.000387	9.82E-05	Downregulated
A0A498J2L6	EF-hand domain-containing protein	0.000388	0.000311	Downregulated
B0YQF6	Small ribosomal subunit protein uS13m	0.000404	0.002943	Downregulated
A0A498ICT6	Uncharacterized protein	0.000406	0.008319	Downregulated
A0A498HUY4	O-fucosyltransferase family protein	0.000415	0.008118	Downregulated
A0A498IS77	VQ domain-containing protein	0.000417	0.04208	Downregulated
A0A498II17	RRM domain-containing protein	0.000428	0.000584	Downregulated

Note: Based on FC values, only the top 15 up-regulated and top 15 down-regulated differential proteins are shown in the table, $p < 0.05$.

S3.5.3. GO Functional Enrichment Analysis

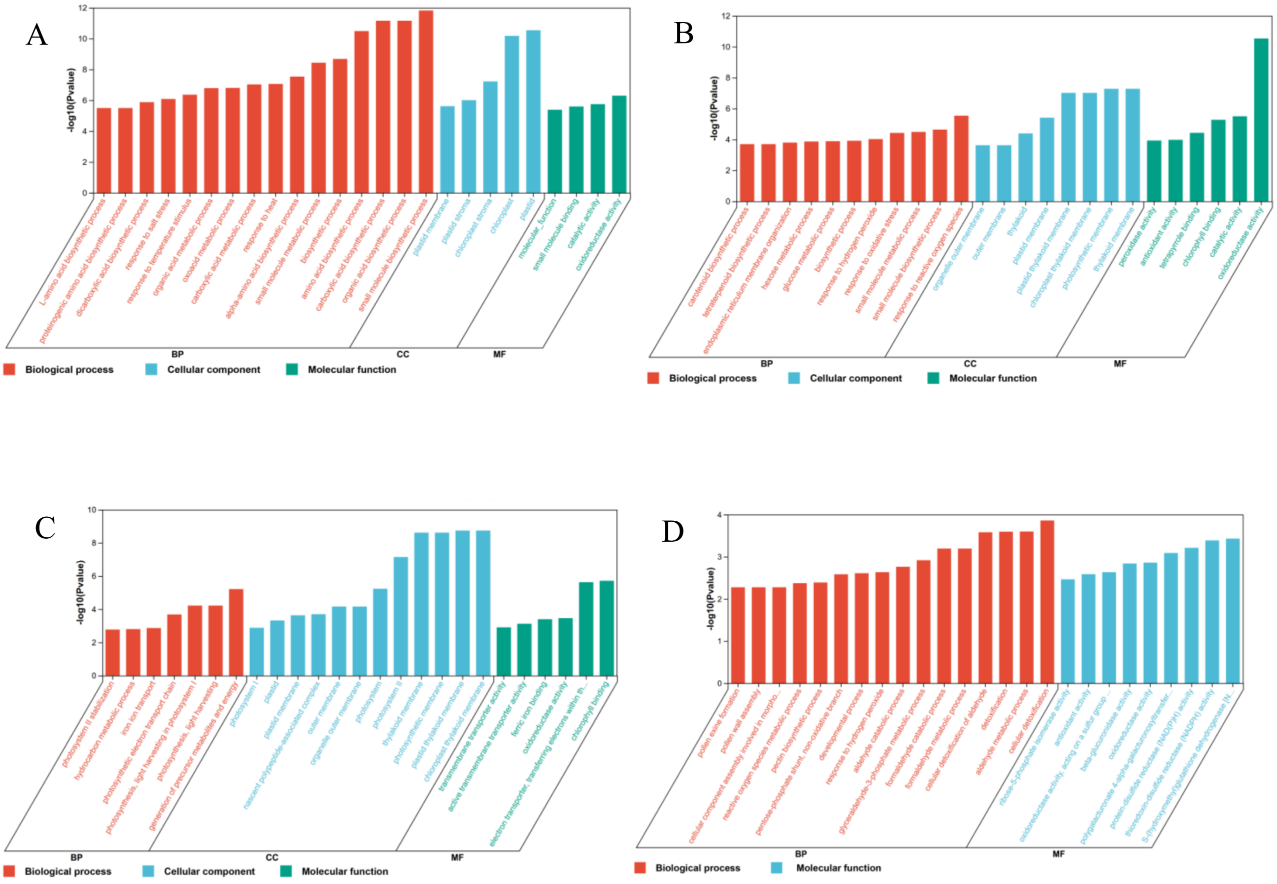

E

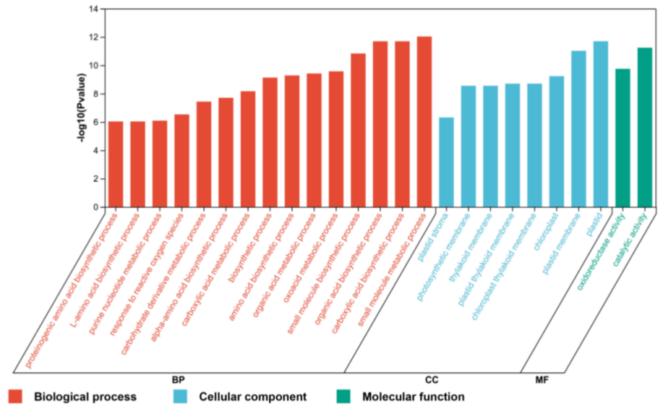

Figure S2. GO functional annotation of differential proteins between HO group and CK group(A), WS group and CK group (B), CT group and HO group (C), CT group and WS group (D), CT group and CK group (E).

As shown in Figure S2(A), after GO annotation of the differential proteins between HO group and CK group, a total of 247 GO terms related to biological processes, 39 GO terms related to cellular components and 148 GO terms related to molecular functions are matched. The differential proteins between HO group and CK group are mainly enriched in biological processes such as biosynthesis process, small molecule metabolism, small molecule biosynthesis, amino acid metabolism, etc.; the cellular components mainly involve plastids, chloroplasts, organelle outer membranes; the molecular functions are mainly characterized by catalytic activity, ion binding, oxidoreductase activity. This indicates that high oxygen treatment may change the basal metabolic state of apple tissues and the redox activity related to plastids.

Figure S2(B) shows that after GO annotation of the differential proteins between WS group and CK group, a total of 155 GO terms related to biological processes, 29 GO terms related to cellular components and 89 GO terms related to molecular functions are matched. The enriched terms between WS group and CK group mainly include biological processes such as carotenoid biosynthesis, hexose metabolism, tetraterpene biosynthesis, oxidative stress response, reactive oxygen species response, etc.; at the molecular function level, categories such as peroxidase activity, antioxidant activity, oxidoreductase activity are enriched. This result is in line with expectations, that is, extract treatment delays browning by enhancing free radical scavenging ability and redox homeostasis.

Figure S2(C) shows that after GO annotation of the differential proteins between HO group and CT group, a total of 133 GO terms related to biological processes, 28 GO terms related to cellular components and 97 GO terms related to molecular functions are matched. The differential proteins between CT group and HO group are enriched in processes such as carbohydrate metabolism, iron ion transport, photosynthetic electron transport chain, photosynthesis, etc., indicating that the combined treatment further adjusts plastid-related metabolism and electron transport network on the basis of high oxygen treatment.

Figure S2(D) shows that after GO annotation of the differential proteins between CT group and WS group, a total of 66 GO terms related to biological processes, 5 GO terms related to cellular components and 52 GO terms related to molecular functions are matched. The differential proteins between CT group and WS group mainly participate in processes such as pectin biosynthesis, hydrogen peroxide response, aldehyde catabolism, detoxification, etc., indicating that the combined treatment may also improve tissue quality through cell wall metabolism and active aldehyde clearance.

Figure S2(E) shows that after GO annotation of the differential proteins between CT group and CK group, a total of 283 GO terms related to biological processes, 32 GO terms related to cellular components and 115 GO terms related to molecular functions are matched. The differential proteins are mainly enriched in processes such as amino acid biosynthesis, purine nucleotide metabolism, reactive oxygen species response, α-amino acid biosynthesis, organic acid metabolism, etc., which further proves that the combined treatment has a systematic impact on the basal metabolic network and oxidative stress response.

S3.5.4. KEGG Pathway Enrichment Analysis

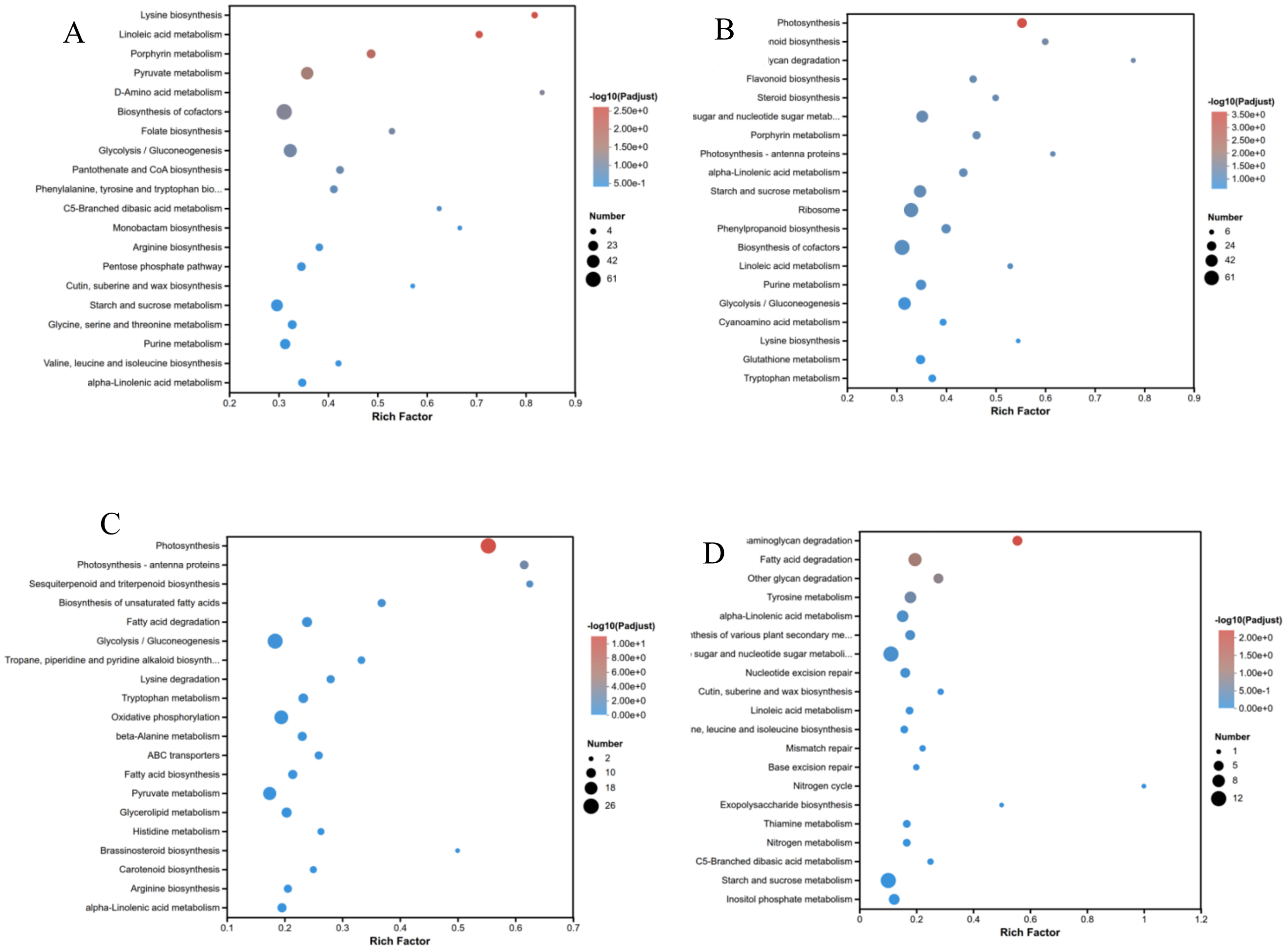

E

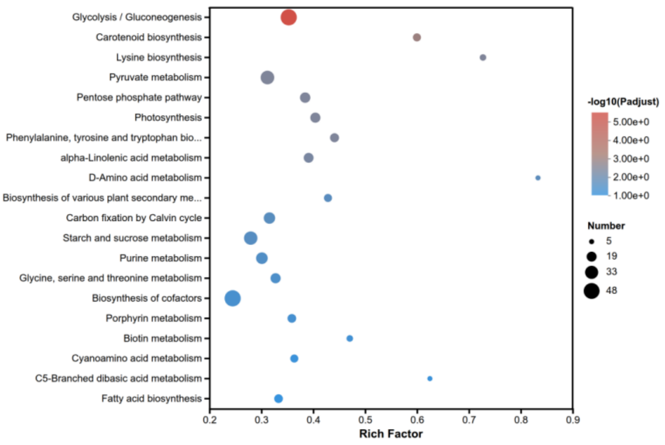

Figure S3. KEGG enrichment pathway analysis of differential proteins between HO group and CK group (A), WS group and CK group (B), CT group and HO group (C), CT group and WS group (D), CT group and CK group (E) .

Note: Rich factor represents the enrichment rate, which is calculated by dividing the number of proteins enriched in a certain pathway (Protein number) by the total number of annotated proteins in this pathway (Background number). The higher the ratio, the higher the enrichment degree. The bubble size corresponds to the number of proteins enriched in the KEGG pathway, and the bubble color represents the Padjust value (corrected p value, Padjust ≤ 1). Only the top 20 pathways with the highest enrichment degree are shown.

Figure S3(A) shows that the differential proteins between HO group and CK group are enriched in a total of 128 metabolic pathways, mainly including biosynthesis of cofactors (map01240), glycolysis/gluconeogenesis (map00010), pyruvate metabolism (map00620), starch and sucrose metabolism (map00500), pentose phosphate pathway (map00030), α-linolenic acid metabolism (map00592), phenylalanine/tyrosine/tryptophan biosynthesis (map00400), lysine biosynthesis (map00300), folate biosynthesis (map00790). These results indicate that high oxygen treatment may reshape the distribution of carbon flux and the pattern of energy supply, thereby affecting membrane lipid oxidation and the generation of stress signals.

Figure S3(B) shows that the differential proteins between WS group and CK group are enriched in a total of 131 metabolic pathways, mainly including biosynthesis of cofactors (map01240), ribosome (map03010), glycolysis/gluconeogenesis (map00010), starch and sucrose metabolism (map00500), amino sugar and nucleotide sugar metabolism (map00520), pyruvate metabolism (map00620), purine metabolism (map00230), photosynthesis (map00195), phenylpropanoid biosynthesis (map00940), glutathione metabolism (map00480), α-linolenic acid metabolism (map00592), porphyrin metabolism (map00860), pentose phosphate pathway (map00030), galactose metabolism (map00052), tryptophan metabolism (map00380). This indicates that extract treatment can not only regulate basal metabolism, but also improve protein turnover efficiency and antioxidant defense capacity. Among them, glutathione metabolism is particularly critical, which may be related to reactive oxygen species clearance and quinone reduction during the browning process of fresh-cut fruits and vegetables.

Figure S3(C) shows that the differential proteins between CT group and HO group are enriched in a total of 118 metabolic pathways, mainly including photosynthesis (map00195), glycolysis/gluconeogenesis (map00010), oxidative phosphorylation (map00190), pyruvate metabolism (map00620), fatty acid degradation (map00071), tryptophan metabolism

(map00380), lysine degradation (map00310), sesquiterpenoid and triterpenoid biosynthesis (map00909). These results indicate that under the background of high oxygen treatment, the combined treatment further regulates the processes of energy conversion and lipid turnover.

Figure S3(D) shows that the differential proteins between CT group and WS group are enriched in a total of 102 metabolic pathways, mainly including glycosaminoglycan degradation (map00531), fatty acid degradation (map00071), other glycan degradation (map00511), tyrosine metabolism (map00350), α-linolenic acid metabolism (map00592), biosynthesis of various plant secondary metabolites (map00999), amino sugar and nucleotide sugar metabolism (map00520), nucleotide excision repair (map03420). These results indicate that the combined treatment further affects cell wall metabolism, lipid metabolism and damage repair processes on the basis of the effect of extract treatment.

Figure S3(E) shows that the differential proteins between CT group and CK group are enriched in a total of 126 metabolic pathways, mainly including glycolysis/gluconeogenesis (map00010), biosynthesis of cofactors (map01240), pyruvate metabolism (map00620), starch and sucrose metabolism (map00500), purine metabolism (map00230), pentose phosphate pathway (map00030), photosynthesis (map00195), glycine/serine/threonine metabolism (map00260), α-linolenic acid metabolism (map00592), glutathione metabolism (map00480), phenylalanine/tyrosine/tryptophan biosynthesis (map00400), glycerolipid metabolism (map00561), cutin, suberine and wax biosynthesis (map00073). From the perspective of anti-browning mechanism, the enrichment of pentose phosphate pathway and glutathione metabolism is particularly critical: the former provides NADPH for cells, while the latter directly participates in peroxide clearance and reduction of oxidation products, and the two jointly provide important support for maintaining redox homeostasis. The combined treatment increases the expression levels of proteins related to these pathways, which is expected to reduce oxidative stress, thereby slowing down the oxidation of phenolic substrates and the browning process.
